# Destabilization of Eukaryote mRNAs by 5′ Proximal Stop Codons Can Occur Independently of the Nonsense-Mediated mRNA Decay Pathway

**DOI:** 10.3390/cells8080800

**Published:** 2019-07-31

**Authors:** Barbara Gorgoni, Yun-Bo Zhao, J. Krishnan, Ian Stansfield

**Affiliations:** 1 Institute of Medical Sciences, University of Aberdeen, Foresterhill, Aberdeen AB25 2ZD, UK; 2 Department of Chemical Engineering, Centre for Process Systems Engineering, Institute for Systems and Synthetic Biology, Imperial College London, South Kensington Campus, London SW7 2AZ, UK

**Keywords:** translation, mRNA decay, mathematical modelling, translational regulation, *Saccharomyces cerevisiae*

## Abstract

In eukaryotes, the binding of poly(A) binding protein (PAB) to the poly(A) tail is central to maintaining mRNA stability. PABP interacts with the translation termination apparatus, and with eIF4G to maintain 3′–5′ mRNA interactions as part of an mRNA closed loop. It is however unclear how ribosome recycling on a closed loop mRNA is influenced by the proximity of the stop codon to the poly(A) tail, and how post-termination ribosome recycling affects mRNA stability. We show that in a yeast disabled for nonsense mediated mRNA decay (NMD), a *PGK1* mRNA with an early stop codon at codon 22 of the reading frame is still highly unstable, and that this instability cannot be significantly countered even when 50% stop codon readthrough is triggered. In an NMD-deficient mutant yeast, stable reporter alleles with more 3′ proximal stop codons could not be rendered unstable through Rli1-depletion, inferring defective Rli1 ribosome recycling is insufficient in itself to trigger mRNA instability. Mathematical modelling of a translation system including the effect of ribosome recycling and poly(A) tail shortening supports the hypothesis that impaired ribosome recycling from 5′ proximal stop codons may compromise initiation processes and thus destabilize the mRNA. A model is proposed wherein ribosomes undergo a maturation process during early elongation steps, and acquire competency to re-initiate on the same mRNA as translation elongation progresses beyond the very 5′ proximal regions of the mRNA.

## 1. Introduction

During expression of genetic information into protein in a eukaryote cell, the primary mRNA transcript is processed and exported into the cytoplasm for translation by the cellular ribosomes. The amount of translated protein produced through gene expression is affected by multiple forms of control, for example at the level of the translational efficiency of the mRNA, and through regulation of protein stability. However, key elements of control exist earlier in the pathway, where the balance of rates of mRNA transcription and decay determine the steady state level of the mature mRNA transcript. mRNA degradation rates are defined by features intrinsic to a given transcript, but mRNA stability can also be dynamically regulated in response to changes in environmental conditions and signaling (reviewed in [1,2]).

Eukaryote mRNAs are modified by a methyl-7-guanosine cap at the 5′ end, and a 3′ poly(A) tail. mRNA stability is imparted by both these elements, in combination with the eIF4 cap-binding complex, and poly(A)-binding protein PABP. These mRNP complexes impart stability by protecting the mRNA from exonucleases that act in a 5′–3′ direction (Xrn1p in yeast; [3]) or in a 3′–5′ direction (the exosome RNase complex; [4]. Stability is further enhanced by mRNA 5′–3′ interactions, the so-called closed loop model of translation. In yeast, PABP binding at the 3′ end interacts with the eIF4G component of the eIF4F cap binding complex at the 5′ end of the mRNA, creating a pseudo-circular mRNA molecule that enhances stability and translational efficiency [5,6].

Despite these important stabilizing interactions, mRNAs are turned over with the gradual removal of A residues from the poly(A) tail over the lifetime of an mRNA through the action of de-adenylases such as the Ccr4-Not1 complex [7,8]. This shortening process gradually removes PABP binding sites, until the point where PABP can no longer bind, exposing the 3′ end to exosome activity, and breaking 5′–3′ mRNA interactions that stabilize the mRNA. mRNA decapping then results, directed by the decapping proteins Dcp1 and Dcp2 [9]. The exposed mRNA 5′ end is then a target for Xrn1-mediated 5′–3′ exonuclease activity [10]. This canonical mRNA decay process, deadenylation-dependent decay, operates alongside other surveillance pathways of transcript turnover which target aberrant mRNAs; no-go decay targets stalled ribosomes [11], non-stop decay target mRNAs lacking an in-frame termination codon [12], and nonsense-mediated decay targets mRNAs that contain a premature in-frame stop codon, a pathway dependent on the Upf1 protein [13,14].

The rate of removal of the poly(A) tail is determined by a complex interplay of mRNA structure [15], mRNA codon composition [16], the nature of the 3′ untranslated region, including AU-rich element binding sites for factors such as the Puf proteins that can stabilize or destabilize mRNAs [17], and the translational efficiency of the mRNA. Of key importance for the latter is the cross-talk between translation and mRNA stability, mediated by links between the translation apparatus and the Poly(A) binding protein (Pab1p in yeast). These are instrumental in maintaining 5′–3′ mRNA interactions and protection of the cap and poly(A) tail, thus modulating mRNA stability. Those interactions involve the Pab1p-eIF4G interactions [5], but also interactions between the translation termination factors and Pab1p. Stop codons are recognized by the essential eRF complex comprising eRF1 and eRF3, a GTPase that enhances termination efficiency [18,19,20]. The release factor eRF3 interacts with Pabp1 [21,22], an interaction that competes with Pab1p-deadenylase interactions, thus regulating mRNA stability [23,24]. The eRF1-eRF3 heterodimer does not simply terminate translation and mediate poly(A) tail interactions, but also acts as a binding hub for proteins such as Rli1 (ABCE1), essential for eukaryotic ribosome recycling [25]. Rli1 itself interacts with a subunit of the eIF3 initiation factor [26]. It seems likely that the action of ribosome recycling could reinforce mRNA 5′–3′ interactions, protecting the cap and thus maintaining stability of the mRNA, although as yet there is no direct evidence of Rli1 impact on mRNA stability beyond a proposed role in no-go decay [27].

The role of the release factor complex in mRNA stability is central to the nonsense-mediated decay (NMD) surveillance mechanism, whereby a premature termination codon (PTC) renders the mRNA unstable via a deadenylation-independent decay mechanism [10,13]. Central to this process appears to be the distance between the PTC and the 3′ end of the mRNA. Increasing the length of the 3′UTR can cause an mRNA to become an NMD substrate, and mRNAs with upstream open reading frames can be NMD-regulated [28,29]. NMD requires the Upf1 protein, which interacts with both eRF1 and eRF3 [14,30]. The release factor complex thus detects the termination codon, with the Upf1 protein acting as a kinetic sensor of PTC position relative to the 3′ end. An attractive model for the mechanism proposes that Upf1p and Pab1p compete for eRF3 binding, with Pab1p-eRF3 interaction suppressing NMD. Consistent with this, tethering Pab1p on the mRNA immediately downstream of an early stop codon will suppress NMD [31], although the mechanism may be more complex, with evidence that it is Pab1p recruitment of eIF4G, rather than eRF3 that suppresses NMD [32]. Intriguingly however, it is known that in mammalian cells, very AUG-proximal stop codons are resistant to NMD [33,34]. A model has been proposed whereby Pabp-eIF4G interactions on the 40S ribosomal subunit are maintained during 5′UTR scanning, and during the early stages of elongation, in part through slow decay of eIF3 association with the small subunit [34]. If there is an AUG-proximal stop codon, it is proposed that association of poly(A) binding protein with an elongating ribosome in the immediately post-initiation phase allows it, rather than Upf1, to preferentially interact with eRF3 at the termination codon, protecting the mRNA from NMD [34]. Many of the models are however undermined by the observation that NMD can nevertheless be triggered in the absence of poly(A) binding protein [35], underlining the mechanistic uncertainty in this area.

The fact that very AUG-proximal stop codons are resistant to NMD is an exception to an expected NMD-controlled behavior, which thus reveals new insight into the process of maturation of a post-initiation ribosome during the early stages of translation elongation. It is however unclear how ordinary mRNA decay is affected by spacing of the termination apparatus and the 3′ end of an mRNA, and how translation termination and Rli1-mediated ribosome recycling might impact on the process of canonical, non-surveillance mRNA decay. The effects of proximity of the termination codon to the poly(A) tail on mRNA stability are therefore unclear. In this study we use NMD-deficient yeast mutants to examine the effects of stop codon position on mRNA decay, placing premature termination codons at increasing distances from the poly (A) tail of yeast mRNAs. Mathematical modelling of translation, including ribosome recycling and mRNA decay, is deployed alongside experimental validation of model predictions to probe the role of juxtaposition of termination codon and poly(A) tail. We show that in the absence of functional nonsense-mediated decay, the control of mRNA stability is robust to all but the most radical alterations in stop codon placement, but that extreme 5′, AUG-proximal stop codons render an mRNA unstable. This instability cannot be significantly ameliorated by stop codon readthrough, which directs a significant flux of ribosomes to the normal position stop codon, and does not appear to be caused by altered efficiency of ribosome recycling via Rli1. We propose that mRNA stability is influenced by ribosome recycling, through early-terminating ribosomes being unable to form mRNP-stabilizing conformations, thus shortening mRNA half-life. The model is predicated on two related mechanisms, either that following translation initiation, ribosomes require successive rounds of translation elongation to become competent to confer mRNA stability via recycling at the stop codon, so early stop codons are destabilizing. Alternatively, early stop codons, by virtue of their physical proximity to the 5′ end, prevent the formation of a stabilizing mRNP complex involving release factors and Pab1p.

## 2. Results

### 2.1. An Early Stop Codon Destabilizes the PGK1 mRNA in A Nonsense-Mediated Decay Independent Manner

The release factor eRF3 and poly(A) binding protein functionally interact on eukaryote mRNAs, linking translation termination to the process of poly(A) tail shortening and mRNA stability. In turn, poly(A) binding protein also interacts with eIF4G as part of a 3′–5′ closed loop interaction between the two ends of an mRNA, linking the process of translation termination to ribosome recycling. However, the interactions between the translation release factor apparatus, Rli1p and the poly(A) binding protein/poly(A) tail complex in mediating mRNA stability are not well understood.

One approach to study this process would be to create a physical separation between the stop codon and the 3′ of the mRNA, by moving the stop codon to a more 5′ location on the mRNA, and examining mRNA turnover rates. However, it is well established that creating early, 5′ stop codons in mRNAs is destabilizing, triggering nonsense-mediated decay mediated by the Upf factors. This problem can be overcome by examining mRNA stability in a *UPF1* deletion genetic background.

We therefore created a series of reporters of mRNA turnover using a galactose-regulated *GAL1* promoter to control transcription of the yeast phospho-glycerate kinase *PGK1* gene. *PGK1* transcription activated by growth on galactose-containing medium could be switched off by the addition of glucose to the growth medium, which inactivates the *GAL1* promoter [36]. A series of *PGK1* alleles was created containing a premature termination codons (PTC) at codons 22, 142, 225 and 319 [37]. In every case we re-engineered the *PGK1* alleles to use only TAA termination codons as the PTCs, ensuring that any difference detected in mRNA stability was due to the position of the stop codon, rather than the identity of the stop codon itself.

The Upf1 protein is central to the nonsense-mediated mRNA decay process, interacting with the release factor eRF1-eRF3 complex to recognize premature stop codons. To test the effect of the premature stop codons in a genetic background in which nonsense mediated decay is disabled, a *upf1* deletant strain was therefore required. However, it has recently been reported that a *upf1* deletant background causes pleiotropic effects with a composite phenotype. Stop codon readthrough is enhanced in a Upf1-deletant, because the mRNA encoding the Alr1p magnesium ion transporter, which contains uORFs in its 5′UTR, is stabilized in a *upf1*-deletant background. The resulting increase in magnesium ions enhances stop codon readthrough [38]. In order to remove this confounding effect of altered magnesium ions on stop codon readthrough, a yeast strain was created carrying a *upf1* deletant, and in which the *ALR1* uORFs had also been deleted, stabilizing the magnesium ion levels. Combining both a *upf1* ablation and *ALR1* uORF removal was crucial to allow us to examine the effect of stop codon introduction without the confounding effects of magnesium ion-driven stop codon readthrough.

**Figure 1:**
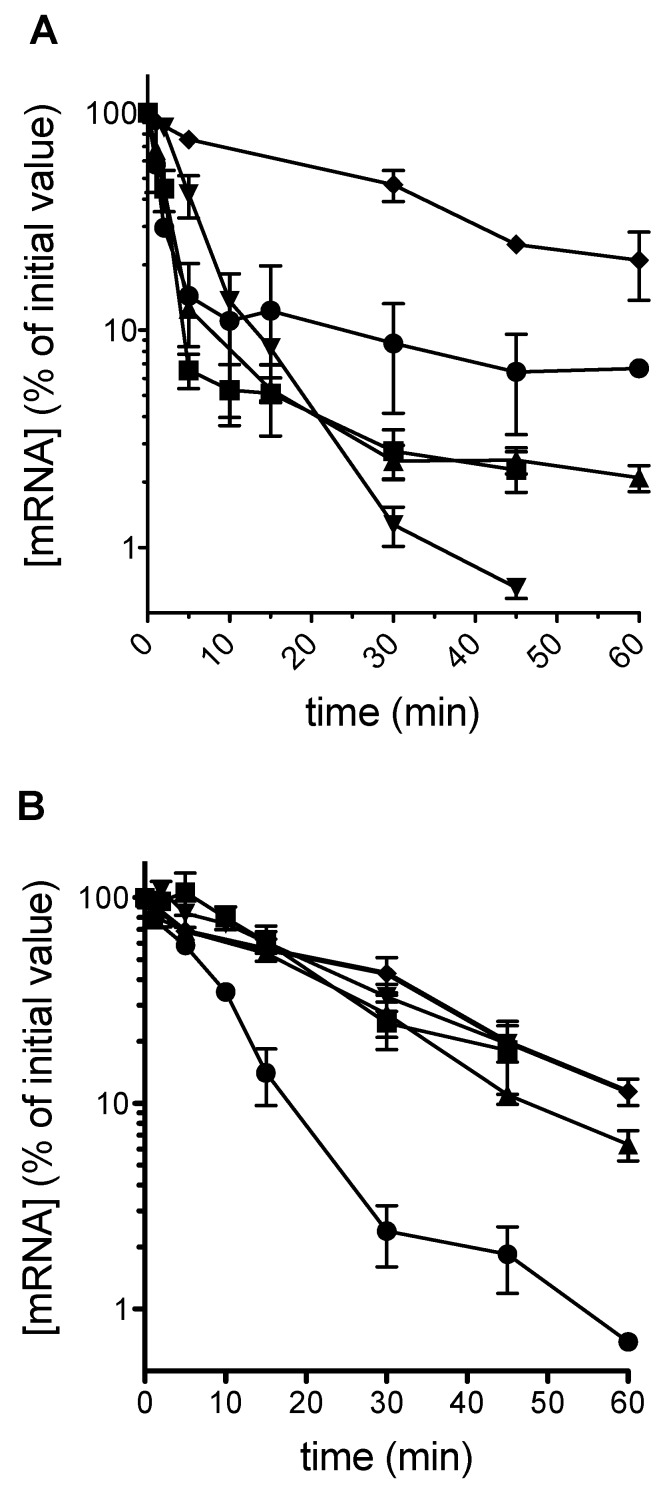
An early stop codon destabilizes the PGK1 mRNA in a nonsense-mediated decay independent manner. The mRNA stabilities of a panel of *PGK1* reporter mRNAs were measured in a wild-type (*UPF1*, panel **A**) or nonsense-mediated decay defective (*upf1*, panel **B**) genetic background. The *PGK1* reporter mRNAs contained a single UAA premature stop codon at one of codon positions 22 (circle), 142 (square), 225 (triangle) or 319 (inverted triangle), and were compared against the wild-type *PGK1* allele (diamonds). Measurements were made in independent biological replicates (*n* = 3, ± standard deviation).

Plasmid-borne *PGK1* PTC alleles were transformed into both a wild-type yeast, and into the *upf1* uORF-less *alr1* strain described above, henceforth termed Δ*upf1*. In a population of cells growing on galactose as a carbon source, and in which the *PGK1* alleles are actively transcribed, a glucose shut-off protocol [3] was used to rapidly switch off transcription. mRNA decay was monitored over time using qRT-PCR. The results show that as expected, in a wild-type background with a functional nonsense-mediated decay apparatus, early position stop codons destabilized the mRNA, with mRNA half-lives significantly lower than those of the normal mRNA (Figure 1A). However, in the Δ*upf1* strain, all mRNAs were stabilized to wild-type mRNA half-life levels of between 15 and 20 min, with the exception of the position 22 termination codon (C22; Figure 1B), with a half-life of 5.6 min, indicating a stop codon-dependent, mRNA destabilization mechanism independent of Upf1p action for 5′ proximal stop codons.

### 2.2. A Theoretical Model Framework to Probe mRNA Destabilization at Early Stop Codons

In order to test hypotheses that would explain these observations, a modelling framework was developed that incorporates a description of translation, with the presence of premature stop codons, along with the possibility of ribosomal recycling onto the same mRNA after termination at both premature and standard position termination codons. In addition, the model incorporates the poly(A)-tail shortening mechanism mediated by Pab1p. Supported by experimental observations, in the model this process is regulated by initiation i.e., initiation events exert a stabilizing influence on the mRNA, and slow the rate of poly(A) removal.

While further details about the modelling are presented in the Methods Section and Supplementary Material (Section 2), we outline a few basic aspects of the model here, as the model is used in multiple places in the results below.

The goal of the model is to provide a mathematical representation of basic hypotheses about the system, which can be used for interrogating system behavior and providing new insights. The model developed is focused on the basic questions at hand: the interplay between basic processes of translation, along with poly(A) removal, as well as recycle. The translation is described via a standard lattice-hopping (TASEP-like) model employed in the literature; this is used in this case by describing the mRNA as a number of coarse-grained segments. Initiation, elongation and termination are described in standard ways. Additionally, the potential of recycle from premature and regular termination codons is incorporated. As mentioned above, the poly(A) shortening mediated by PABP, and its dependence on initiation is incorporated.

With these aspects, the structure of the model is defined. The parametrization of the models is discussed in the Methods Section and parameters defined in the Supplementary Information (Section 3.1). In addition to a standard parameterization of the translation steps (with initiation limiting, and elongation rates equal, and equal to regular termination rate constant), the key extra parameters were the recycle propensity, as well as the termination rate constants at the premature stop codon. The recycle propensity is chosen to reflect basic hypotheses about recycle (discussed in the context of the results), while we contrast two cases of premature termination rate constants, one which is equal to regular termination, and the other which is smaller by a factor of 10. The latter case results in the propensity of ribosomes to queue up near the premature termination codon, and allows the simulation of a queueing regime.

The models are analyzed both deterministically (mean field analysis via an ODE model) and stochastically. The key point is that the results from the model, reflecting basic qualitative trends, follow transparently from the interplay of basic factors and the structure of the model.

We now use the model to investigate the behavior observed experimentally above.

**Figure 2:**
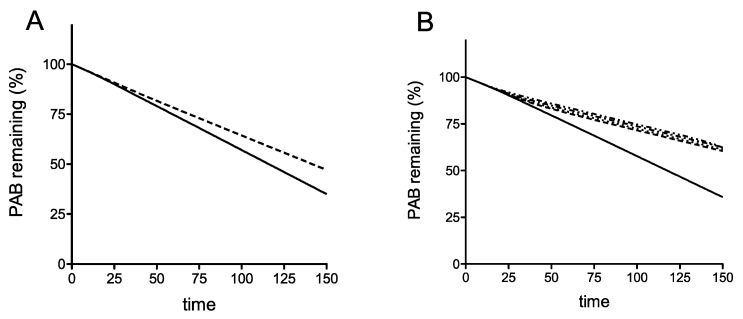
A deterministic model of translation including ribosome recycling can simulate the destabilization of an mRNA carrying an early stop codon, but not a late stop codon. (**A**) The position of the premature stop codon can play an important role in mRNA stability, indicated by the proportion of poly(A) binding protein remaining over time: the earliest stop codon (relative position 2; in the sequence of coarse-grained segments in the model, corresponding to the codon 22, solid line) has a reduced stability compared to the other four stop codon positions (Relative positions 12, 19, 27, 33 in the sequence of coarse-grained segments in the model; dotted lines, curves identical and therefore superimposed). In this iteration of the model, there is no recycle of ribosomes at the premature stop codon. (**B**) Introducing a propensity of the ribosome to recycle preserves the trends observed in panel A, although the effect of the early stop codon is accentuated, and there is a faster decay rate of the position 2 stop codon relative to the mRNA variants with a later position. A recycle propensity of 0.1 was employed for codon 2 (solid line) and 0.9 for all others (dashed lines).

Using the model simulations, when the termination rate at the premature termination codon (PTC) is slow, we find interestingly, that even in the absence of recycling, there is a clear difference in mRNA stability for the earliest PTC, compared with the other four more 3′ proximal PTCs (Figure 2A). Interestingly, if the PTC had a termination rate constant comparable to that of regular termination and elongation (these constants assumed equal), the stability characteristics of the different locations of the PTC are practically identical as studied in the ODE model (Figure S5). This already points to a subtle interplay between premature termination rate and location of the PTC. The reason for this behavior is that a low termination rate allows for queueing of ribosomes, close to the initiation codon, thus effectively preventing de novo initiation. Building on this, in the case where the termination rate at the PTC is low, we see that introducing a sharply increasing and saturating recycling propensity (lower for the earliest PTC and equal for the other 4, reflecting a basic hypothesis of reduced recycle propensity for early PTC: see Methods) only accentuates this reduced stability of the earliest PTC. Taken together, we can say that a reduced stability of the earliest PTC can occur through a combination of location (when the termination rate at the PTC is low, allowing for queueing which reduces new initiation) and reduced recycle, which also reduces initiation through the recycle pathway. Each of these factors by themselves contribute to a clear reduction in stability. Using these assumptions, this deterministic model of translation including ribosome recycling can simulate the destabilization of an mRNA carrying an early stop codon, but not a late stop codon.

### 2.3. Stop Codon Readthrough Is Insufficient to Restore Complete Stability to the Codon 22 Premature Stop Codon Variant mRNA

Premature termination codons (PTCs) are known to destabilize mRNA via NMD, but mRNAs containing PTCs can be stabilized via stop codon readthrough. The arrival of a ribosome at the natural stop codon stabilizes a PTC-containing mRNA, in part due to relocation of mRNA-associated Upf1 to the 3′UTR, particularly important in organisms that exhibit NMD, but whose genes have few introns. We wished to investigate whether a flow of ribosomes to the natural stop codon would stabilize the PTC mRNAs in the Δ*upf1* background, by allowing a flow of ribosomes to reach the natural stop codon. We therefore explored whether directing stop codon readthrough would stabilize an mRNA with an early (codon 22) stop codon in the Δ*upf1* background.

Initially, the *SUP4* UAA suppressor tRNA was used to drive readthrough of the codon 22 UAA codon. The effect of *SUP4* stabilization was compared for the codon 22 UAA codon in a wild-type background, as well as in a Δ*upf1* background. In order to control for any plasmid transformation effects, the *SUP4* tRNA gene was flanked by a *tetO* operator sequence, which allowed the doxycycline-regulated expression of *SUP4* tRNA [39]. We expected to see that the *SUP4* tRNA would stabilize the mRNA in a cell with a functioning NMD pathway. This stabilization effect could therefore be compared with the effect of the same tRNA on the codon 22 mRNA in a Δ*upf1* background.

**Figure 3:**
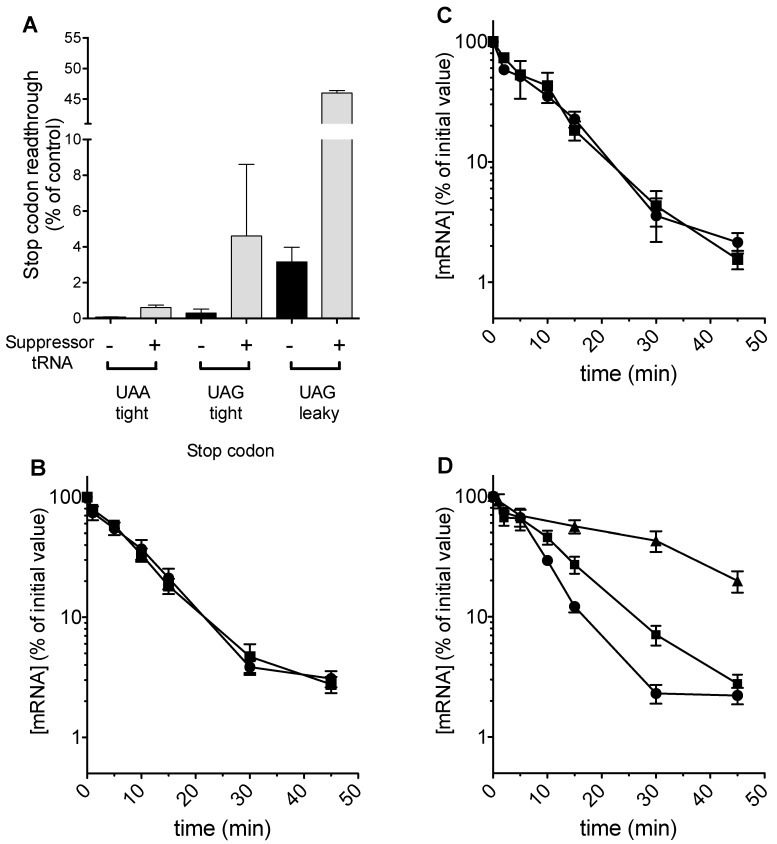
Stop codon readthrough is insufficient to restore stability to the C22 early premature stop codon variant mRNA. (**A**) In a Δ*upf1* yeast strain, stop codon readthrough of either UAA or UAG codons was measured using a dicistronic reporter assay, with the stop codon inserted at the junction of an in-frame lacZ-luciferase translational fusion. Stop codon readthrough was measured either in the presence or absence of a UAA, or a UAG suppressor tRNA (Sup4^Tyr^_UUA_ [bars 1 and 2], or Sup65^Gln^_CUA_ [bars 3–6] respectively). The stop codon was placed either in a good stop codon context (‘tight’, bars 1–4) or a leaky Tobacco Mosaic Virus context flanked by a CAA triplet on either side (‘leaky’, bars 5,6) using the same context as the various engineered codon 22 contexts in the *PGK1* mRNA stability reporter. Measurements were made in independent biological replicates (*n* = 3, ± standard error). (**B**–**D**) The mRNA stability of the *PGK1* reporter carrying a codon 22 premature stop codon was measured in the presence (filled squares) and absence (filled circles) of a suppressor tRNA. Graphs show mRNA stability of a reporter with a UAA tight stop codon 22 in the presence and absence of a UAA suppressor tRNA (**B**), reporter stability with a UAG tight stop codon 22 plus and minus a UAG suppressor tRNA (**C**) and reporter stability with a leaky UAG stop codon with and without a UAG suppressor tRNA; for reference the stability of the wild-type *PGK1* reporter is shown (closed triangles, **D**). Measurements were made in independent biological replicates (*n* = 3, ± standard error).

As predicted, tRNA expression could be regulated using doxycycline, and readthrough of a UAA stop codon placed in a good context was measured at 2% in the presence, but not absence of 5 μg mL^−1^ doxycycline (Figure 3A). However, despite detectable levels of stop codon readthrough, no effect of stabilization on codon 22-UAA mRNA was detected in the Δ*upf1* background (Figure 3B). Examination of the codon 22 UAA stop codon context did not reveal any of the known nucleotides which render a yeast stop codon leaky, for example a C nucleotide in the +1 position.

We therefore sought to test whether stop codon identity was relevant, using another tRNA that drives high levels of stop codon readthrough, a UAG-cognate glutamine tRNA^Gln^_CUA_ which our previous work had shown to be efficient at directing UAG readthrough [40]. In order to deploy this tRNA, the codon 22 stop codon in the *PGK1* allele was changed to TAG in a good context. The tRNA was expressed from a single copy plasmid, and although it directed readthrough at 4.6% (Figure 3A), there was again no detectable alteration to mRNA half-life in a Δ*upf1* background (Figure 3C).

Finally, the codon 22 TAG context was engineered so that the TAG stop codon was flanked with a downstream CAA codon, part of the TMV readthrough signal [41]. This change rendered the UAG termination codon leaky, and readthrough at 3% was detected in a wild-type cell. This increased to 46% in the presence of the tRNA^Gln^_CUA_ (Figure 3A). Directing significant levels of readthrough in this way increased the half-life of the codon 22-TAG CAA *PGK1* allele, but only to the extent of a 1.5 fold stabilization in the Δ*upf1* background and failing to restore mRNA stability to wild-type allele levels (Figure 3D). In contrast, expression of the codon 22 allele along with the same tRNA in a wild-type strain with functional NMD increased the half-life of the mRNA more than 3 fold (data not shown).

In conclusion, stop codon readthrough was capable of only partially stabilizing mRNAs with very early premature stop codons, at codon 22, both in a wild-type and Δ*upf1* background. Notably, readthrough of 46% was required to exert any detectable effect on mRNA stability of the codon 22 PTC variant in both a *UPF1* wild-type and Δ*upf1* backgrounds. Moreover, the readthrough-stabilizing effect was much less marked in a Δ*upf1* background than in a wild-type cell with a functional NMD pathway.

**Figure 4:**
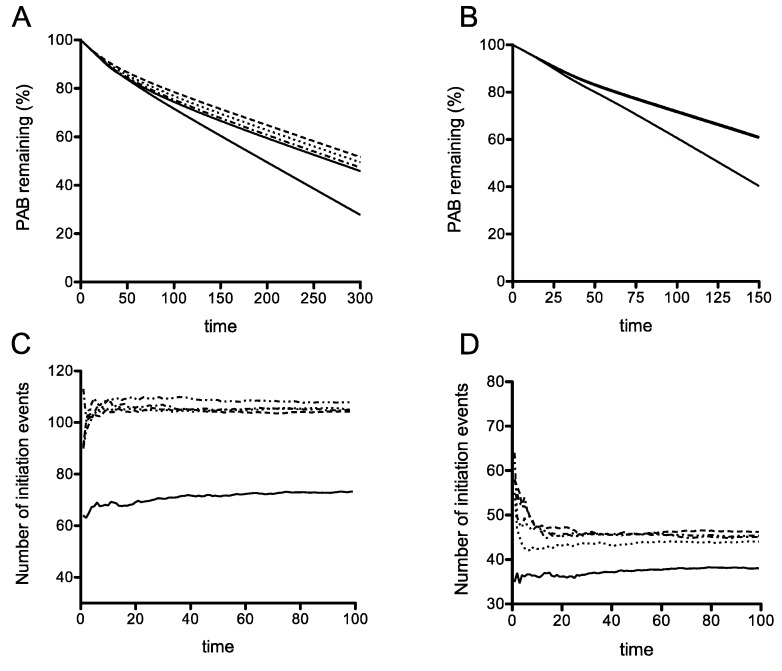
Model simulation of stop codon readthrough effects. (**A**) Simulation of translation and mRNA decay using an initial framework where ribosomal queuing is not permitted. Shortening of the poly(A) tail, and thus proportion of bound poly(A) binding protein is used as a proxy for mRNA stability. Elongation and regular termination rate constants 10 times higher than the de-novo initiation rate constant and the total rate constant at the premature termination codon were used. The readthrough propensity used, 0.5, does not result in significant stabilization of the C22 early PTC variant (solid, lower line). (**B**) Simulation of translation and mRNA decay using similar parameters as panel A, but with a framework where ribosomal queuing is now permitted. A readthrough propensity of 0.9 was used, while keeping the total rate constant at the premature termination codon fixed. Note that decreasing the total rate constant at the premature stop codon can accentuate the queueing effect. The solid lower line represents the C22 allele. (**C**,**D**) Stochastic simulations show similar patterns of stability. In each case, the solid, lower line represents the stability plot for the early, codon 22 proxy stop codon. A recycle propensity of 0.1 was used for the codon 22 proxy, with a 0.9 recycle propensity used of all other premature termination codons. Simulation of a non-queuing regime was performed with 50% stop codon readthrough (**C**) or with a queuing regime and 90% readthrough (**D**).

The effects of increasing stop-codon readthrough were then studied directly in our model. The simulations showed that the differential instability associated with the earliest PTC is maintained even when the readthrough at this PTC is increased significantly, even as high as 90% (Figure 4B). This result was obtained using a simulation where rates of translation initiation had the propensity to form queues. It was therefore of interest to contrast this result in a queueing regime simulation, with one in a non-queueing regime, where the termination rate constant at the PTC is the same as that at a regular TC (and also the same as the elongation rate). Here a sharp gradation in recycling can result in a reduced stability of the earliest PTC, and increasing readthrough even up to 50% efficiency can maintain this reduced stability (Figure 4A). These results which were obtained from the ODE model are also seen in stochastic model as well (Figure 4C,D). Taken together, the model indicates that a reduced stability of the early PTC arising from a confluence of a slow PTC and reduced recycle propensity is remarkably robust to changes in readthrough.

### 2.4. Interrupted Recycling via Rli1 Activity Is Not a Destabilizing Influence at a Premature Stop Codon

Following translation termination, yeast ribosomes are recycled via the activity of the ATPase Fe-S cluster protein Rli1 (ABCE1 in mammals). Rli1 interacts with the release factor complex eRF1-eRF3 [42], and the translation initiation factor eIF3. In parallel, Pab1p, bound to the 3′ poly(A) tail interacts with the initiation factor eIF4G, part of the 5′ cap-binding complex. In this way, Rli1 is likely to play a central role in translation initiation via the closed loop model, and therefore contribute to the maintenance of mRNA stability by promoting translation initiation. The effect of stop codon position on ribosome recycling is unknown, but it was reasoned that the observed instability of the codon 22 allele of *PGK1* in the Δ*upf1* background may be due to a failure to efficiently recycle ribosomes on the mRNA, in turn leading to mRNA destabilization.

In order to test this hypothesis, we sought to examine the effect of defective Rli1 recycling on the codon 225 (C225) *PGK1* allele. In an NMD-deficient background, a *PGK1* allele bearing a PTC at codon 225 was hitherto shown to exhibit the same mRNA half-life as the wild-type *PGK1* allele (Figure 1). Therefore, we tested whether this C225 allele could be rendered unstable by interfering with ribosome recycling through expression of a dominant-negative Rli1 protein. Accordingly, the Δ*upf1* strain IS400/1c was transformed with the PTC mutant C225 *PGK1* allele, and additionally either pBAR7, expressing a wild-type FLAG-tagged *RLI1* allele, or with pBAR8, expressing dominant-negative *rli1*-E493Q (Dong et al., 2004). Both *RLI1* alleles were under the control of a galactose-inducible promoter. Accordingly, cultures were grown to steady-state on galactose-containing medium, and following a shift to glucose to switch off transcription of the *PGK1* PTC allele, *PGK1* mRNA decay measured as described (Materials and Methods). The mRNA half-life of the unstable codon 22 PTC allele was also measured as a control comparator.

**Figure 5:**
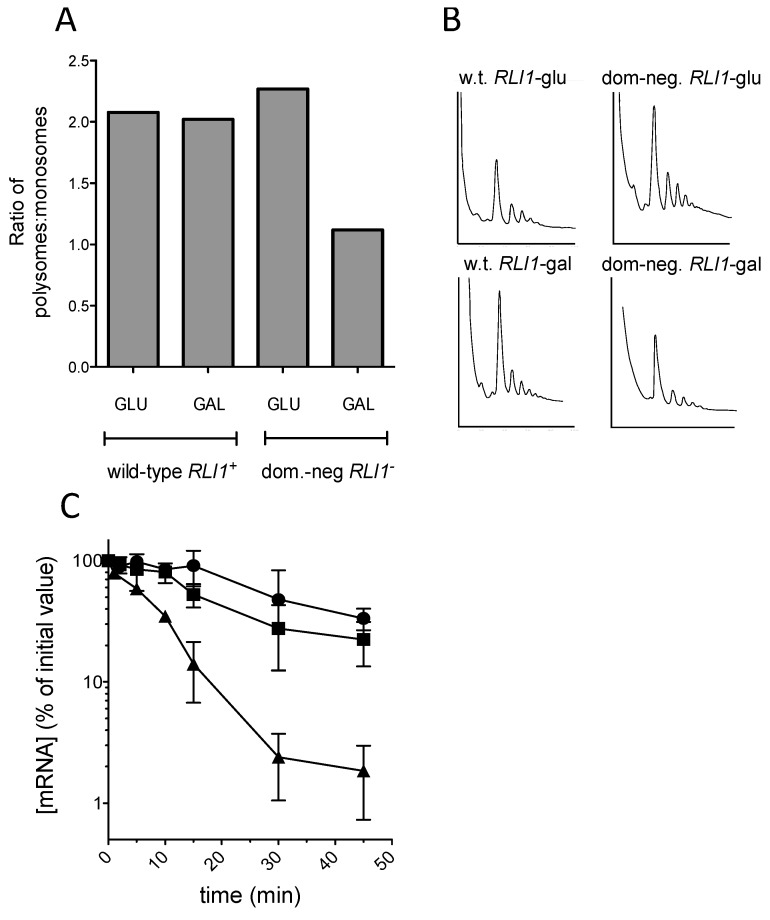
Failure to recycle ribosomes via Rli1 does not destabilize mRNAs. (**A**,**B**) The translation defect conferred by a dominant negative *RLI1* allele was confirmed by carrying out polysome profile analysis on the dominant-negative *RILI1* allele, and *RLI1^+^* wild-type control, both under control of the *GAL* promoter. Polysomes were analyzed in the presence of glucose (Rli1p-repressed) and galactose (Rli1p-induced), confirming a reduction in the polysome:monosome ratio, indicative of a ribosome recycling defect, during galactose-induction of the dominant-negative Rli1p. (**C**) The mRNA stability of a *PGK1* reporter mRNA carrying a premature stop codon at position 225 was measured in a nonsense-mediated decay defective genetic background either in the presence (squares) or absence(circles) of a dominant negative *RLI1* allele. For reference, the mRNA decay profile of the codon 22 *PGK1* allele is presented (triangles). Measurements were made in independent biological replicates (*n* = 3, ± standard error).

The results showed clearly that as previously reported, expression of the *rli1*-E493Q allele caused a significant reduction in the proportion of polysomal ribosomes, thought to be due to defective ribosome recycling following translation termination at a stop codon [26]; (Figure 5A,B). Despite the observed polysomal defect indicative of defective recycling, the stability of the C225 allele was not significantly reduced, and strains expressing FLAG-Rli1-E493Q exhibited similar PTC C225 mRNA decay rates to those expressing FLAG-Rli1p (Figure 5C). The results therefore do not support the hypothesis that impaired recycling of ribosomes via defective Rli1 can drive mRNA destabilization.

### 2.5. Model Simulation of Resumed Scanning by Ribosomes After Early Termination

The premature translation termination codon at position 22 of *PGK1* effectively creates a 22 codon upstream ORF (uORF) on the mRNA. Ribosomal subunits can resume scanning initiation after terminating translation at the uORF stop codon, and upon encountering a downstream AUG methionine codon, re-initiation can take place. Such downstream re-initiation events require the stochastic re-acquisition of a ternary initiation factor complex comprising eIF2, GTP and methionyl-tRNA^Met^ which then enables the now 43S subunit to recognize an AUG codon (reviewed in Hinnebusch, 2005). It was therefore important to incorporate a description of this known ribosomal behavior within the model, to test whether potential downstream scanning after termination at codon 22, and re-initiation at 3′ AUG codons, could have material effects on our prediction that it is impaired ribosome recycling at early stop codons that is responsible for destabilization of the C22 allele mRNA.

**Figure 6:**
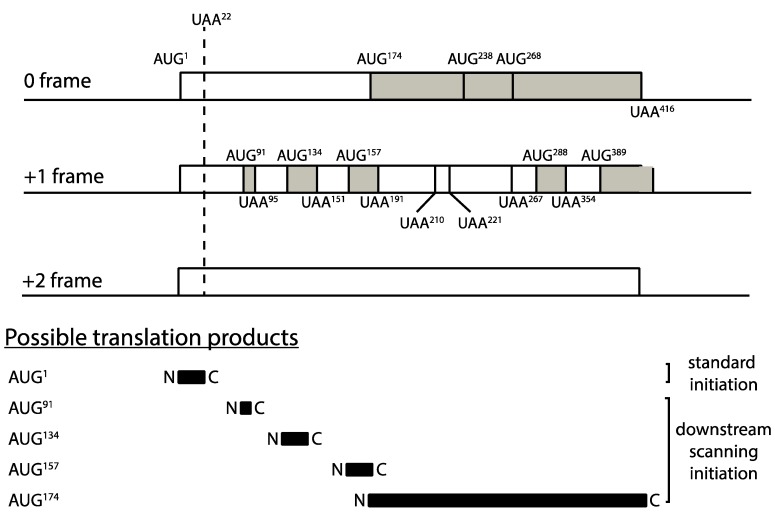
Reading frame structure of the PGK1 mRNA stability reporter indicating the potential for resumed scanning downstream of the codon 22 premature stop codon. The positions of AUG initiation codons downstream of codon 22 are indicated in units of codons, along with the potential open reading frames that they initiate, in each of the three potential translational reading frames.

An examination of the *PGK1* codon 22 allele downstream of codon 22 revealed a complex landscape of AUG codons and short open reading frames, which could theoretically create multiple opportunities for downstream scanning, reinitiation and consequent ribosome recycling (Figure 6). All of these alternative events would compete with, and reduce, recycling opportunities at codon 22, with potential modulatory effects on mRNA stability. There are multiple AUG codons downstream of codon 22, the first three of which are present at codons 91, 134 and 157 for example (all in the +1 frame), and even the most 5′ of these, at codon 91, is at a scanning distance (270 nucleotides) within which ternary complex reacquisition and downstream initiation is possible according to the *GCN4* paradigm [43]. Accordingly, we further adapted our model of translation, ribosome recycling and mRNA stability, to examine the hypothesis that resumed scanning after codon 22 termination in the C22 allele may be the cause of the mRNA instability observed experimentally. The model was used to simulate resumed scanning at stop codons terminating uORFs, and re-initiation at respective downstream AUG codons.

The adapted model therefore represented the series of open reading frames in Figure 6. This model is an augmentation of the basic model of translation we have employed earlier, to incorporate the mechanism of rescan. This allows us to examine the effect of rescan systematically, as a perturbation of the earlier model.

We developed a simplified model to capture the basic aspects of rescan. This was achieved as follows. At the premature stop codon, we now allow for the extra event of the small ribosomal subunit rescanning, downstream of the C22 stop codon at an experimentally measured rate for scanning 43S subunits [44]. At a subsequent AUG initiation codon, this ribosomal subunit will initiate, and a full ribosome will be assembled. Furthermore, this ribosome will undergo translation elongation until an-in-frame stop codon is encountered, where again termination will occur (with the possibility of recycle), but with the additional possibility of rescanning. Our model monitors the progression of both ribosomes and ribosomal subunits (at distinct rates), with strict exclusion enforced (see Supplementary Material for details). Downstream start and stop codons are located proportionally (to their actual codon location described in Figure 6) in our computer simulation. Overall, in our simplified model, we allow for the possibility of one further re-initiation and one additional premature termination. The rate constant associated with this additional termination codon is assumed to be the same as that at the first premature stop codon. The rate of scanning of the 43S ribosomal subunit is incorporated, noting the relative rates of progression of a ribosomal subunit and a regular ribosome.

The mechanism of rescan essentially allows for a fraction of terminating ribosomes to engage in a rescanning process. In the model, a terminating ribosome can follow two pathways: a recycle pathway and a release to the pool of initiating ribosomes, or, if termination occurs at a short ORF, resumed scanning. When we incorporate the latter rescan, we assume that a fixed fraction of ribosomes being released into the pool are involved in rescan. To serve as a contrast, we also examine the opposite case, where the rescan “draws” from recycle, i.e., a fraction of ribosomes which may have otherwise been recycled are involved in rescan (discussed below). Further details for modelling ribosomal rescanning are presented in Figure S4. Probing the model (in relation to a model without rescan, used above) highlights the effects of this perturbation.

**Figure 7:**
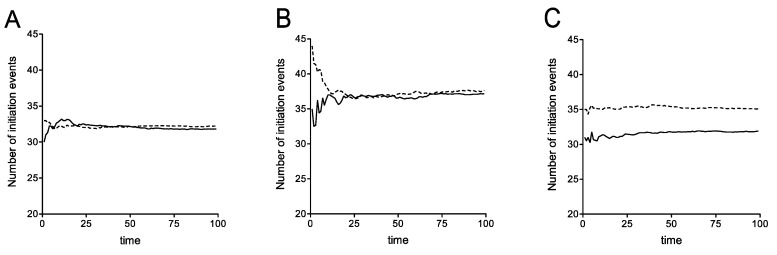
Stochastic simulations of a downstream scanning model of translation. Stochastic simulations of a downstream scanning model of translation that simulates resumed scanning after C22 termination. This figure explores the effect of rescan (solid line) at the earliest stop codon, contrasted with the case of no rescan (dotted line), using stochastic simulations. In all cases shown a queuing regime is simulated. (**A**) Queuing regime with no recycle; (**B**) Queueing regime with high readthrough; (**C**) Queueing regime: Rescan directly borrows from recycle.

The details of the computational (stochastic) model incorporating rescan are presented in Supplementary Material. We have computationally studied this expanded stochastic model and the essential results are shown in Figure 7. As seen in Figure 7A, where recycle is disabled, the possibility of ribosomal rescanning results in a small percentage change in the number of initiation events indicating that ribosomal rescanning by itself does not have a substantial effect on mRNA stability. This effect is seen, even with recycle, and also with recycle where the readthrough propensity at the premature stop codon was increased at the level described previously (Figure 7B). This implies that the net effect of the rescan is a small increase in stability, by funneling a fraction of ribosomes (which would otherwise be released into the pool) to the 5′ end, some of which get recycled. Finally Figure 7C demonstrates a scenario where rescanning happens at the expense of recycle: In this case, rescanning itself has a clear (though moderate) destabilizing effect, simply because this reduces the recycle. Note that the rescanning ribosomes, either make it through to the regular stop codon, or terminate at more 5′ stop codons, and in both cases, a fraction of these do not get recycled.

Taken together, noting the distinct trends of stability of the early PTC and the later ones presented in Figure 2, we conclude that introducing ribosomal rescanning still results in a clear difference in mRNA stability associated with this early PTC at codon 22. Furthermore, if rescanning occurred at the expense of recycle, this can in fact accentuate the reduced mRNA stability associated with this early PTC. This consolidates the conclusion that incorporating the possibility of ribosomal rescanning does not fundamentally change the principal conclusion, that even if ribosomal rescanning does occur, it would not affect the stability of the C22 PGK1 allele.

## 3. Discussion

There are now multiple strands of evidence that indicate a 5′–3′ mRNA interaction during eukaryote translation. Poly(A) binding protein interacts with eIF4G, the scaffold protein, which is part of the cap-binding eIF4F complex [5,6]. In yeast the translation termination factor complex of eRF1 and eRF3 interacts with the poly(A) binding protein, mediated by eRF3 [21,22]. Post-termination the ribosome is released from the mRNA by the ribosome recycle factor Rli1p (yeast)/ABCE1 (mammals) to participate in new rounds of translation. Rli1 interacts with components of the translation initiation factor complex eIF3, bound to the 43S pre-initiation complex [25]. These studies contribute to a picture of an interaction between the translation process, and poly(A)-binding protein at the 3′ end, with translation contributing to the regulation of poly(A) tail shortening rate, and thus mRNA half-life. Some recent reports have cast doubt on the simplistic description of an essential 5′ cap-3′ tail interaction that promotes translatability, showing such interactions may be non-essential [45], or are not supported by 5′–3′ proximity measurement [46]. Nevertheless, a significant body of evidence supports termination complex-3′ end interactions [21,22], and it should also be remembered that 5′–3′ interactions may operate in trans to concatenate mRNAs.

How translation and mRNA decay function as a linked molecular process is however unclear. Blocking translation initiation increases the rate of mRNA turnover in eukaryotes [3,47,48]. In addition to these observations, there is evidence for and against the interaction between the release factors and Pab1p having an effect on mRNA turnover in yeast [23,24,49]. Blocking the flow of ribosomes during elongation using cycloheximide also stabilizes mRNAs [50]. There are thus conflicting results concerning the effects of translation on mRNA stabilization. Nevertheless, it is clear from the interactions between the key proteins that the two processes are tightly linked, although a ‘unifying model’ that explains the interplay between mRNA turnover and translation at a molecular level is still required. In seeking to develop that unifying model, exceptions to expected molecular biological behavior will likely be instructive. For example, it has been reported that premature termination codons (PTCs) at the very 5′ of an open reading frame are resistant to nonsense-mediated decay (NMD) driven by the Upf1p NMD factor [33,34]. It is suggested that 5′–3′ Pabp-eIF4G interactions on the 40S ribosomal subunit persist during and beyond 5′UTR scanning, to include the early stages of elongation [34]. This lasting interaction may compete with proposed poly(A) binding protein-Upf1 interactions needed to designate an early stop codon as a PTC [34]. This example thus describes a failure of Upf1-driven NMD due to a very 5′-proximal situation of a stop codon. In this work we describe another very distinct departure from expected molecular process, namely that *in the absence of Upf1p* and therefore the absence of a functional NMD apparatus, very 5′ proximal stop codons highly destabilize an mRNA, whereas other more 3′ PTCs show identical stability to that of a wild-type mRNA.

It is well established that premature stop codons destabilize an mRNA via the NMD pathway, and that there is a polarity effect with early, 5′ proximal stop codons being more destabilizing, although it is now clear that very early 5′ PTCs are an exception to this rule [33]. In our study, we employed a series of *PGK1* alleles carrying PTCs at identical positions to those of a previous study [37], and examined their stability in wild-type, and NMD-deficient yeast. As expected, the data showed clearly that in the wild-type background, PTCs were destabilizing, and as reported by Cao and Parker, the more 5′ PTCs (e.g., codon 22) were more destabilizing than the more 3′ proximal PTCs (codon 319; Figure 1A). Interestingly we saw no evidence that the early codon 22 PTC was NMD-resistant. In β-globin PTC studies in mammalian cells, PTCs at codon 15 were NMD-resistant, but those at codon 39 NMD-sensitive [33].

In this work, following deletion of *upf1* to ablate NMD, testing of the same family of PTC-containing *PGK1* alleles revealed that as expected the stabilities of all but one of the PTC alleles were now restored to the levels of those of a standard transcript lacking a PTC. The exception was the C22 allele, which was as unstable as in the wild-type yeast with functional NMD (Figure 1B). It is clear that the early positioning of the PTC at codon 22 had in some way rendered the mRNA unstable, but using an NMD-independent mechanism. Supporting our observation and analysis, a reduced steady-state level of codon 22 mRNA in a *upf1* background has been previously experimentally shown, although its causes not analyzed, [35,37], and is explained here by our measurement of its reduced half-life in a *upf1* background.

To provide insight into this phenomenon, we developed a mathematical model of the coupled translation-mRNA decay process as a hypothesis-testing tool. The TASEP model represents the translation process of initiation, elongation and termination, followed by ribosome recycling and re-initiation of the model ribosomes on the same mRNA. A limited set of assumptions was employed, the first of which is that translation initiation exerts a stabilizing influence on poly(A) tail shortening, reflecting research that shows blocking initiation destabilizes mRNAs by increasing decapping rates [3,47,51]. Second, the model describes a variable reinitiation propensity depending upon PTC position, allowing the testing of our hypothesis that the 5′ proximal PTC at C22 may be destabilizing because in some way ribosomes are prevented from recycling and re-initiating following termination. The analysis revealed that the model can replicate the mRNA-destabilizing effect of an early, codon 22 stop codon, while leaving the half-lives of all other PTC-containing mRNAs identical to wild-type (Figure 2). That replication of the experimentally observed half-lives could be achieved using the model in two ways. The first was by slowing the rate of termination in the model, causing ribosome queues that then reduced the rate of initiation, thus destabilizing the mRNA (this effect is pronounced for early PTCs). One point worthy of note regarding any queueing is that it could potentially cause No Go Decay (NGD) independent of Upf1 and Rli1. We point out that our model does not incorporate NGD, and in fact queueing at a premature stop codon and activating the NGD apparatus could occur at any premature stop codon location, whereas we show here a C22-specific stop codon effect. We additionally ensured all our PTC stop codons were in similar ‘termination factor-favorable’ contexts, minimizing chances of pausing.

Moreover, in the original paper by Doma and Parker identifying NGD they triggered this decay using a very strong and long, translation elongation-inhibiting stem loop with a 31 base pair stem [11]. They then show that although premature termination codons (PTC) can trigger NGD, the PTC effect is extremely weak, and barely detectable relative to the canonical NGD triggered by the strong stem loop. This observation, combined with our use of efficient stop codon contexts, make it extremely unlikely that NGD is the primary cause of the destabilization. In our work, we can only say that the C22 mRNA is unstable, and we are not able to offer specific predictions on the mechanism of its destabilization.

Moreover, we know of no reason to expect that C22 termination should be slower than any of the other PTCs, with its UAA stop codon in an apparently good termination context with no known readthrough determinants [52]. The high efficiency of C22 stop codon recognition was in fact experimentally confirmed using the dicistronic readthrough reporter assay, indicating efficient recognition by the release factor complex (Figure 3A). Using the model simulation, the second, and only other way of simulating instability of the PTC codon 22 (C22) mRNA was by assuming that the propensity of ribosomes terminating at the PTC to reinitiate on the same mRNA was lower than is the case for the other PTCs. This reduces the rate of C22 translation initiation and exerts a destabilizing influence on the C22 mRNA relative to the other PTC mRNAs (Figure 2). Lastly, although the simulations focused on the non-queueing regime (for reasons discussed), the queueing regime reveals similar trends.

To understand the mechanism of this NMD-independent destabilization, plasmid-borne suppressor tRNAs were used to direct readthrough of ribosomes through the premature stop codon at position 22, and consequential effects on mRNA stability monitored in an NMD-deficient background. We hypothesized albeit that suppressor tRNAs are relatively inefficient at driving readthrough, small numbers of ribosomes arriving at the natural termination codon might have a catalytic, licensing effect on mRNA stability. In fact, generating 4% readthrough had no stabilizing effect on the C22 mRNA, and even readthrough efficiencies of 46% using a leaky UAG stop codon and a strong suppressor tRNA only partially stabilized the C22 mRNA. This implied a bulk flow of ribosomes to the natural stop codon was required to completely restore mRNA stability (Figure 3B–D). The inference is that whatever stabilizing influence termination at the natural stop codon is having it may be operating in competition to the destabilizing influence of the C22 termination events. Simulating stop codon readthrough using the mathematical model could also successfully replicate the resistance of C22 mRNA instability to readthrough events, and in the model, even 90% readthrough could not full stabilize C22 mRNA. The presumed inability to recycle ribosomes, originating from the C22 PTC, into the initiating pool therefore must confer instability on the mRNA, despite significant frequencies of stop codon bypass. Alternatively, the participation of a C22 PTC in a termination codon-5′ cap mRNP may in some way destabilize the poly(A) tail-cap interactions.

We speculated that the ribosome recycling factor Rli1 may be playing a role in the mRNA stabilization process; recycling of ribosomes will channel them to the cap structure to initiate, a known mRNA stabilization process [3,47,51]. If that recycling process is compromised, perhaps when termination occurs at a 5′ proximal PTC, mRNA instability may result, such as seen at PTC C22. We hypothesized that if Rli1 recycling were compromised, it may render ordinarily stable mRNAs with 3′ proximal downstream stop codons (C225) unstable. However, despite using a dominant negative form of Rli1, no significant mRNA destabilization could be triggered through use of dominant negative Rli1 alleles expressed in trans. Although this result does not exclude the involvement of the recycling process in determining mRNA stability, it seems likely that Rli1 does not play a leading role.

It is known that short open reading frames can permit small subunit resumed scanning downstream of a stop codon, with uORFs as long as 105 nucleotides permitting resumption of scanning [43,44]. The codon 22 stop codon thus terminates a short ORF of 66 nucleotides well capable of permitting ribosomes to resume scanning downstream. The next PTC tested, at codon 125, is too long for this to occur. In that sense, of the PTCs tested in this study, C22 has distinct properties. It was unclear how resumed scanning of ribosomes downstream of the C22 PTC might influence mRNA stability. We therefore used the model framework we had established to test the hypothesis that mRNA destabilization resulted when ribosomes terminate at C22, then resume scanning for a downstream AUG codon, and translate a downstream complex landscape of very short uORFs, and longer ORFs (Figure 6). Some of the ORFs downstream of the C22 PTC were spaced far enough apart to allow ternary complex re-acquisition after C22 scanning. By applying the model to analyze these multiple downstream outcomes, we were able to test whether the model would predict C22 allele mRNA stability, since resumed scanning events would potentially push the point at which ribosomes would recycle at downstream stop codons further towards the 3′ end. However, the model predictions showed clearly that despite the included assumption of resumed scanning events into a simulation of C22 mRNA turnover, there was no material alteration to its predicted half-life, and in the new simulations the half-life remained significantly shorter than that of the other modelled PTCs. Using model simulation, we therefore concluded that any putative downstream scanning events after C22 termination could not explain the observed instability of the PTC-22 mRNA in a *upf1* background.

**Figure 8:**
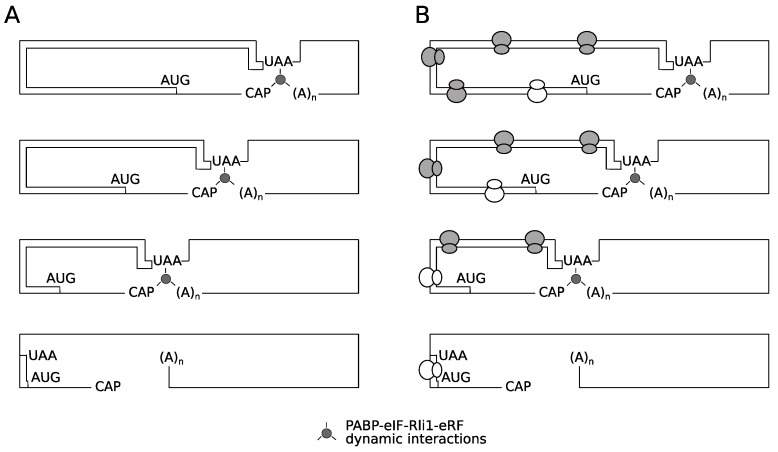
A spatio-mechanical model and temporal-biochemical model of PTC position effects on non-NMD instability. Two models for the effects of an early (codon 22) stop codon on the stability of an mRNA. Assuming an absence of a nonsense-mediated decay apparatus, in panel (**A**), spatio-mechanical constraints limit the ability of the terminating release factor-rRli1 recycling factor complex to interact with a closed-loop eIF-PABp complex, thus destabilizing the mRNA. An alternative model is presented in panel (**B**); ribosomes translating immediately post-initiation are not competent to either recycle on the same mRNA following termination, or in some way to promote mRNA stabilization during the course of the normal elongation/termination cycle. That stabilization property is acquired over the course of a number of translation elongation cycles, allowing ribosomes that have terminated at more 3′ stop codons to promote mRNA stabilization, while termination at early (e.g., codon 22) termination codons cannot impart stability.

The results presented clearly show the destabilizing effect of a premature termination codon, in the absence of functional NMD. However, there was no evidence that this occurs through impaired activity of Rli1 on recycling of ribosomes. Nevertheless, it remains possible that ribosomes terminating at very early stop codons are structurally unable to recycle. Two models are proposed to explain the unusual behavior 5′ proximal PTCs as exemplified by the C22 allele (Figure 8). The first is what we term a spatio-mechanical model. In essence, a very 5′ termination codon in some way physically prevents the assembly of a functional recycling complex involving the ribosome, the poly(A) tail-Pab1p complex, and the release factors-Rli1 complex. This might be due to the mechanical properties of the mRNA reducing the chances that a functional 5′ (stop codon)-3′(mRNA tail) pseudo-circular mRNA-protein complex emerges to successfully channel ribosomes back to the 5′ end. The instability of this presumed 5′–3′ interaction would in turn destabilize the PTC-22 mRNA. However, we consider this model formally possible, but fairly unlikely, for the following reason. It is well established that mRNA is a flexible molecule capable of forming hairpin loops with loop sizes as small as 4-6 nucleotides [53,54]. The 5′ untranslated region of the *PGK1* mRNA used here is 44 nucleotides [55], representing a total length of 110 nucleotides between the 5′ cap and the C22 stop codon. We regard it as most likely that this length poses no stearic hinderance barriers to the interaction of cap-binding proteins with the C22 stop codon region of this premature termination codon transcript.

It is also possible that ribosomes terminating at very early stop codons are functionally unable to recycle, perhaps because of initiation factors remaining bound to elongating ribosomes. Ribosomes elongating immediately after an initiation event appear to have special properties, perhaps due to bound factors, that decay as elongation progresses. For example while uORFs permit resumed scanning (termination codon context permitting; [43] and downstream re-initiation, longer uORFs cannot promote resumed scanning of ribosomes [44]. Other evidence is suggested by the NMD-resistance of very early PTCs, which been attributed to the association of an eIF4G-Pab complex with elongating ribosomes, that only persists in regions of the ORF immediately downstream of an initiation codon. Therefore we propose a second, biochemical-temporal model, where it is proposed that after translation initiation, the ribosome is functionally unable to recycle until sufficient numbers of elongation events have taken place, to allow either loss of (initiation) factors that prevent ribosomal recycling, or gain of later stage elongation factors that license ribosome recycling (Figure 8). The identification of the translation factors involved in any post-initiation, ‘*maturation-during-elongation*’ process is beyond the scope of this study, but could in principle utilize doxycycline-regulated translation factor shut-off strains (e.g., [56]). Our identification of the rapid turnover of mRNAs with 5′ proximal premature termination codons, in an NMD-independent manner provides new evidence that post-initiation ribosomes have properties that distinguish them from a later-stage elongating ribosome. This study therefore contributes to the development of a unifying model of translation integrated with mRNA turnover.

## 4. Materials and Methods

### 4.1. Mathematical Modelling of Translation

The goal, from the modelling perspective, is to provide a framework for the investigation of the non-NMD based destabilization mechanisms to connect with experimental results. We develop a modelling framework appropriate to the goal at hand. The modelling framework, in its most basic form, incorporates the description of translation, with the presence of premature stop codons, along with the possibility of ribosomal recycle after termination and release. In addition, it incorporates the poly(A)-tail shortening mechanism mediated by PABP, and the regulation of this by translation initiation. Additional extensions of the basic modelling framework to describe ribosomal rescanning, will be described subsequently, in the context of the relevant results.

#### 4.1.1. Modelling Translation

A variety of approaches have hitherto been used to model translation [57,58,59,60,61,62]. Building on these approaches, our modelling approach involves the deployment of a basic framework describing translation (further details, and equations are presented in Supplementary Material, Section 2). For modelling translation, we employ a standard TASEP-like model, which simply incorporates the initiation, ribosomal progression on the mRNA (elongation) as well as termination, at both the regular and premature stop-codons. The level of detail is maintained appropriate to the goals and focus of the study. Thus, we do not describe detailed initiation or termination complexes, or indeed auxiliary biochemical steps involved in elongation. We employ both mean field (which involves ordinary differential equations, henceforth referred to as ODE) models as well as stochastic descriptions as part of our study. Both approaches are widely used in the literature. The former (which makes a mean-field approximation in the TASEP model) is easier and more transparent to analyze, while the latter incorporates features such as strict exclusion at a codon and allows for a study of stochastic effects. While our focus is not on stochastic aspects as such, using the two descriptions in tandem allows for a broad platform for the investigation of such systems.

#### 4.1.2. Translational Recycle

In addition to the basic translation modelling, we incorporate the effect of recycle. This is described by having a terminating ribosome, recycle back, with a specific probability to the initiation site (assuming it is vacant). Our framework incorporates the possibility of recycle from both premature and regular stop codons.

#### 4.1.3. Modelling Poly(A)-Tail Shortening

Since NMD is deactivated, the determinant of mRNA stability is the poly(A) tail shortening. Our modelling framework, incorporates in a concise way, a basic assumption about the inverse correlation between initiation frequency and stability; the higher the initiation rate, the lower the PAB removal and consequently the longer the time for poly(A) tail shortening. Within the model, poly(A) tail shortening recapitulates a well-established experimental phenomenon e.g., [37], is thus used as a simple proxy for destabilization of an mRNA. This is incorporated in both the mean field description (incorporating a deterministic ODE description of the poly(A) tail shortening) and well as in the stochastic description (basic stochastic model of poly(A) tail shortening: see Supplementary material).

#### 4.1.4. Model Parameterization

Once the basic model structures are defined, the only remaining factors involve the parameterization. We again make multiple simplifying assumptions related to this. We assume that all elongation rate constants are the same, as is the termination rate constant at the regular codon. We assume that the initiation rate constant is a factor of ten smaller than the elongation rate constants. This is consistent with initiation being a rate limiting step of protein synthesis.

With regard to the termination rate constant at the premature stop codon, we examined two cases, from the viewpoint of model analysis: (i) The termination at the premature stop codon is a factor of ten smaller than that at the regular stop codon (ii) the termination at the premature stop codon is equal to that at the regular stop codon. This is examined briefly in the Supplementary material and we show that the predictions from such a model make an interesting contrast with case (i) and consequently the experimental results as well.

Our framework allows us to examine the effect of recycle propensity as well. In certain cases, in the results, we examine the effect of absence of recycle from premature stop codons. One of the basic cases we examine corresponds to reduced recycle propensity for very early PTCs, which is studied by incorporating a sharp gradation in recycle propensity with the premature stop codon position: this is mathematically realized in the model by describing the recycle propensity as a sharply increasing and saturating function which shows very little dependence on stop codon position, beyond a certain location. In our model for the locations of PTCs considered, this results in a recycle propensity in the earliest PTC a factor 1/9 less than the other PTCs (the results we obtain do not depend on specific numbers, but instead only depend on the significantly reduced recycle propensity at the early PTCs). Our results point to effects in case (i) which occur independent of recycle, but which can be further accentuated by this gradation in recycle propensity. In our simulations, we employ 35 codons/segments, as a coarse-grained description of the system (see Supplementary Material for further discussion of methods).

#### 4.1.5. Robustness of the Results

Our analysis of the model has been performed in two distinct parameter regimes, resulting in specific predictions which were tested and confirmed. The results of the model arise from a specific combination of effects, which we are able to dissect and analyze. As such, the model plays a role of compactly encapsulating these effects (recycle, initiation-limitation, location of codons, connection to (Poly(A)-tail shortening) and drawing conclusions therefrom. Moderate changes to model parameters (which basically do not severely distort the essential interplay of factors) also lead to similar conclusions for the same reason.

This is further exemplified by our incorporation of ribosomal rescanning; incorporation of these distortions to the basic model does not alter the essential conclusions.

#### 4.1.6. Ribosomal Rescanning in the Model

The basic TASEP model which is at the core of overall model is a widely employed model of translation. We incorporate a description of ribosomal rescanning (discussed in detail in Supplementary Material, Section 2.3) as an augmentation of the model used earlier. This then allows us to transparently dissect the effect of ribosomal rescanning as a perturbation of the earlier model. This could incidentally also find use in other experimental contexts focusing on ribosomal rescanning.

### 4.2. Yeast Growth Conditions and Media

*Saccharomyces cerevisiae* strains were grown in either YPD complete medium (2% *w*/*v* glucose, 2% *w/v* peptone and 1% *w*/*v* yeast extract), or synthetic defined (SD) medium (0.67% *w*/*v* yeast nitrogen base [Formedium], 2% *w*/*v* glucose, and amino acids and nucleotides as required at 0.02 g·L^−1^ or 0.06 g·L^−1^ in the case of leucine). Liquid cultures were grown at 30 °C, 225 rpm in an orbital shaker.

### 4.3. Yeast Strains

*S.cerevisiae* strains carrying a deletion in the *UPF1* gene exhibit an increased concentration of cytoplasmic magnesium ions [38], which in turn enhances levels of stop codon readthrough. A *UPF1* deletion exerts its effect by stabilizing the upstream open reading frame-containing *ALR1* transcript, which encodes a magnesium ion transporter. A yeast strain was therefore created that carried both a disrupted *UPF1* gene, and an *alr1* allele lacking uORFs in the 5′ untranslated region of its transcript. Accordingly, the *alr1* 5′UTR region, marked by a geneticin resistance kanMX marker, was amplified from strain MJY477, a kind gift from Prof A. Jacobson, University of Massachusetts Medical School, USA; Johansson and Jacobson, 2010); (*MATa ura3 leu2-2 his3-11,15 trp1 can1-100 ade2-1 P_ALR1_::kanMX6-P_ALR1_* (AUG1* AUG2A* AUG2B* AUG3*)-3HA), and integrated into strain BY4741 in place of the wild-type *ALR1* promoter and 5′UTR by homologous recombination. This intermediate strain was mated to strain BY4742 Δ*upf1::NatMX,* the resulting diploid sporulated and tetratype and non-parental ditype tetrads selected, within which were spores that carried both geneticin and nourseothricin resistance markers (i.e., *P_ALR1_::kanMX6-P_ALR1_* (ΔAUG), Δ*upf1*), generating strain IS400/1c. The marker combination was confirmed using diagnostic PCR.

### 4.4. Stop Codon-Containing PGK1 Alleles

A pre-existing series of *PGK1* alleles had been previously created containing a premature stop codon (PTC) at codon at codon position 22 (TAA premature stop codon, or PTC), codon 142 (a TAG PTC), codon 225 (a TAG PTC) and codon 319 (a TGA PTC) [63]. These plasmid-borne alleles are under control of the yeast *GAL1* promoter, transcriptionally active in galactose-containing growth medium, but inactive if glucose is present.

In this work, the identity of the premature stop codon was altered using site-directed mutagenesis so that in every case the PTC was a TAA stop codon. Plasmids pBG142, pBG225 and pBG319, (derivatives of pHD142, pHD225 and pHD319 respectively; [63] express a *GAL1* promoter-controlled *PGK1* allele carrying TAA stop codon at positions 142 (denoted *pgk1*-142^TAA^), 225 (denoted *pgk1*-225^TAA^) and 319 (denoted *pgk1*-319^TAA^) respectively. Mutagenic primers 1099 and 1100 (Table S1) were used to create the position 142 TAA codon, primers 1101 and 1102 (Table S1) to create the position 225 TAA codon, and primers 1103 and 1104 (Table S1) to create the position 319 TAA codon. Two additional plasmids, kind gifts from Dr Roy Parker (Univ of Arizona, USA) similarly expressed P*_GAL1_* controlled *PGK1*, either lacking PTC (pRP469, expressing *PGK1*), or with a TAA stop codon at position 22 (pRP555, expressing *pgk1*-22^TAA^).

Variants of the position 22 stop codon construct were also created using site-directed mutagenesis in which the TAA stop codon was changed to a TAG stop codon to allow readthrough by a UAG-recognising tRNA^Gln^ (plasmid pBAR9, expressing *pgk1*-22^TAG^), or changed to a TAG stop codon followed by CAA to create a poorly-recognized stop codon (plasmid pBAR10, expressing *pgk1*-22^TAG-CAA^), or changed to a TAG stop codon flanked by the Tobacco Mosaic Virus stop codon readthrough context [41] to create a more poorly-recognized stop codon (plasmid pBAR11, expressing *pgk1*-22^TAG-TMV^).

Mutagenic primers 1168 and 1169 (Table S1) were used to create the position 22 TAG codon, primers 1202 and 1203 (Table S1) to create the position 22 TAG-CAA codons, and primers 1204 and 1205 (Table S1) to create the position 22 CAA-TAG-CAA codons in the TMV readthrough context.

### 4.5. Doxycycline Regulated Suppressor tRNA Expression

Suppressor tRNAs were expressed using plasmids carrying cloned copies of either the tyrosine *SUP4^oc^* tRNA with a mutated UUA anticodon, directing UAA readthrough (plasmid pSUP4-tetR, *HIS3 CEN*), or a *SUP70* glutamine tRNA with mutated CTA anticodon (derived from plasmid pH2 [40], directing UAG readthrough (plasmid pBAR12, *HIS3, CEN*). In each case, the tRNA gene was flanked by a *tetO* operator, introduced 7 nucleotides upstream of the tRNA gene transcription start site using PCR, allowing control of tRNA expression by the tetR protein [39]. Both tRNA gene segments (which included 300 nucleotides of 3′ genomic context) were cloned into pRS413 cut with XhoI-SalI.

Following cloning of the tRNA segment, a *GAL*-regulated tet repressor gene (derived from pGalTR1; [39] with additional *PGK1* transcriptional terminator cassette to prevent transcriptional readthrough into the tRNA gene region, was cloned into each of the tRNA vectors at their NotI site, allowing doxycycline regulation of suppressor tRNA expression when transformants were grown on galactose-containing growth medium.

### 4.6. Rli1 Dominant Negative Allele

To engineer galactose-regulated expression of wild-type Rli1p, and dominant-negative (E493Q) Rli1p, vectors pDH201 and pDH203 [26]: A kind gift from Drs J Dong and A. Hinnebusch, NIH) were adapted as follows. Plasmid pDH201, carrying *GAL1* promoter-regulated FLAG-tagged *RLI1*, was digested with ApaI, the overhangs filled with T4 DNA polymerase, and relegated, thus destroying the *URA3* selectable marker, but leaving the *leu2-d*-selectable marker intact. This plasmid was designated pBAR7. A related plasmid, pBAR8 was created by carrying out the same process on plasmid pDH203, which carries *GAL1* promoter-regulated FLAG-tagged *rli1*-E493Q.

### 4.7. Stop Codon Readthrough Assay

Translational readthrough of UAA stop codons was measured using a plasmid-borne dicistronic readthrough assay [64] in which either an in-frame TAA or TAG stop codon was inserted at the junction of a constitutively expressed *lacZ*-firefly luciferase gene fusion. Stop codon readthrough is reported by the ratio of luciferase: β-galactosidase activities, expressed relative to a control construct lacking the in-frame stop codon between the two reporters. The TAA and TAG stop codons were engineered in contexts that either directed efficient termination (6 nucleotides both 5′ and 3′ of the yeast *SUP45* gene; [52]) or leaky termination, derived from the Tobacco Mosaic Virus stop codon readthrough context [41].

### 4.8. Polysome Profile Analysis

Polysome analysis was carried out essentially as described [65]. Yeast strains were grown in SD medium until mid-log phase was reached, and cycloheximide added to a final concentration of 200 μg mL^−1^, 15 min. before harvest. Cells were broken in polysome lysis buffer (25 mM Tris pH 7.4, 50 mM KCl, 5 mM MgCl_2,_ 5 mM 2-mercaptoethanol containing 200 μg mL^−1^ cycloheximide using glass bead lysis, and a Fastprep bead disruptor machine (MP Biologicals). Following supernatant clarifying centrifuge steps (10 min, 3500× *g*, 15 min. 15,600× *g*), lysate was loaded onto a 12 mL 15–50% sucrose gradient made up in polysome lysis buffer, and the gradient spun at 36,000 rpm for 2.25 h using a Beckman SW41 ultracentrifuge rotor.

### 4.9. mRNA Decay Measurement

mRNA decay rates were measured using modifications to a published method [36]. A 200 mL culture of yeast transformed with a plasmid expressing *PGK1* under control of the *GAL1* promoter was grown overnight in SD medium containing 2% *w*/*v* galactose until an optical density at 600 nm of 0.5 was reached. Following cell harvest and resuspension in SD medium lacking carbon source, glucose was immediately added to a final concentration of 2% *w*/*v* to begin the time course. Samples (2 mL) were taken at regular time points, cells harvested in ice-cold tubes, and cell pellets frozen in liquid nitrogen, and stored at −80 °C.

Cell pellets were lysed using glass beads, and RNA purified using a Qiagen RNAeasy kit according to the manufacturer’s instructions. mRNA amounts were quantified using qRT-PCR. Superscript III (Invitrogen) was first used to create cDNA, and the subsequent RT-PCR reaction performed using Roche LightCycler 480 SYBR Green I Master mix in a Roche RT-PCR instrument. Levels of *PGK1* mRNA were monitored using primers 1076 and 1096 (Table S1). *SCR1* RNA was used as a housekeeping, stable structural RNA that acted as a stable reference against which to measure mRNA decay [62], and was monitored using qRT-PCR using primers 1113 and 1114 (Table S1).

## Supplementary Materials

The following are available online at https://www.mdpi.com/2073-4409/8/8/800/s1, Supplementary methods and Table S1: Primers used in this study.
