# Supplementary material for: Destabilization of Eukaryote mRNAs by 5′ Proximal Stop Codons Can Occur Independently of the Nonsense-Mediated mRNA Decay Pathway
Source: Cells. 2019 Jul 31;8(8):800. doi: 10.3390/cells8080800 (PMC6721604; doi:10.3390/cells8080800)
Supplement: Supplementary file 1 [file cells-08-00800-s001.pdf]

Stop codon regulation of eukaryote mRNA stability dependent on position but independent of the nonsense-mediated decay pathway

Barbara Gorgoni[1], Yun-Bo Zhao[2,3], J. Krishnan[2], Ian Stansfield[1*]

[1]School of Medical Sciences, Institute of Medical Sciences, University of Aberdeen, Foresterhill, Aberdeen, AB25 2ZD, UK
[2]Department of Chemical Engineering, Imperial College London, South Kensington, London SW7 2AZ, UK
[3]College of Information Engineering, Zhejiang University of Technology, Hangzhou 310023, China

Email: Y.-B. Zhao - ybzhao@ieee.org; J. Krishnan - J.Krishnan@imperial.ac.uk; I. Stansfield - i.stansfield@abdn.ac.uk;

*Corresponding author

In this supplementary material, we discuss multiple details associated with the models we have employed in the paper. At the outset we note that the modelling framework was geared towards the particular focal point of the paper. In particular, the modelling is aimed at complementing experiments to elucidate key experimental observations:

- An early PTC (premature termination codon) is destabilizing even without NMD;
- Significantly increasing readthrough rate at the early PTC does not significantly alter the destabilization of the mRNA.

Modelling approach: We employ models at an appropriate level of granularity for the questions at hand. Since the focus is on destabilization mechanisms associated with premature termination codons at particular locations, we employ a coarse-grained codon-based description of the mRNA, which is then analyzed deterministically and stochastically. This level of granularity and detail is sufficient for the conclusions we draw, and our consideration of multiple parameter regimes allows us to consolidate the essential insights. We have also performed theoretical analysis for simplifications of models, though that is not discussed here. We then expand our modelling framework to examine the feature of ribosomal rescanning, which needs a dedicated model of its own. These features are described below.

1 Model assumptions

Assumption 1 (Source of mRNA decay)**.** *In the model, there are, in principle, two sources of mRNA decay.*

- *mRNA decays immediately after all poly(A) elements are removed;*
- *Active destabilizing mechanisms can be responsible for mRNA decay.*

In this study the former factor is a key ingredient in destabilization introduced by a premature stop codon.

Assumption 2 (Poly(A) shortening)**.** *Poly(A) shortening is conditional on the PABP removal and some natural intrinsic rate.*

- *Poly(A) shortening is subject to some natural intrinsic rate, but occurs only after the covering PABP is removed;*
- *The rate of the removal of the last PABP is determined by some natural rate and is affected by the total initiation rate. Initiation events slow down PABP removal.*

We now discuss the models employed.

2 The models

The core of all our models involve coarse-grained, codon descriptions of the mRNA translation, upon which is overlaid PABP removal. As discussed in the main text, we particularly focus on the role of ribosomal recycle in this context. We present our modelling approach as a hierarchy of models of increasing complexity: (a) Basic codon-based model without ribosome recycling or rescan (b) Incorporating the effect of ribosomal recycle (c) Modelling of ribosomal rescan. The models are analyzed both through stochastic simulation as well as numerical analysis of the mean-field (deterministic) ordinary differential equation (ODE) equivalent, in cases (a) and (b). The analysis of mean-field models allows for a complementary analysis, both computationally (which can leverage the tools used for analyzing ODEs) and also analytically, with certain simplifications. The analysis of the mean field models provide an estimate of the mRNA decay time. In the stochastic description, each step in the stochastic simulation is a stochastic event of either translation, poly(A) shortening, PABP removal, or mRNA decay, implying that decay is a stochastic event and may occur before any stationary state is achieved.

2.1 Basic codon-based model without recycle nor rescan

We first start by focussing on the basic codon-based model where both recycle and ribosomal rescan are absent. The basic model schematic is illustrated Fig. S1. The model consists of three layers. mRNA decay in the top layer could depend on both the mRNA state varying with the translation process in the middle layer, and the poly(A) shortening in the bottom layer whose PABP removal rate is controlled by the total initiation rate in the translation process in the middle layer. The poly(A) shortening is the main factor for our study.

The translation process depicted in the middle layer is the backbone of the model: its own state could affect mRNA decay, and the initiation rate controls poly(A) shortening which also affects mRNA decay. The figure depicts initiation, elongation (depicted as sequential progression of ribosomes) and termination. Also shown here is a premature termination codon (labelled "s") where the ribosome may terminate. There is a probability that the ribosome may read-through this premature termination codon.

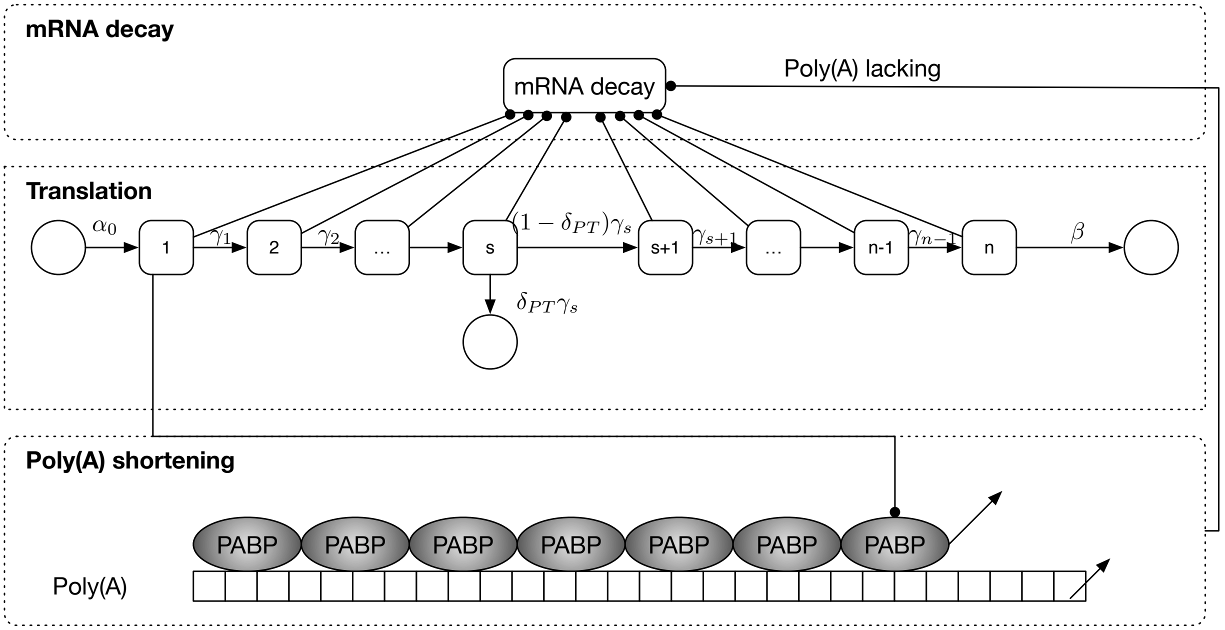

Figure S1: The basic codon-based model structure without ribosome recycle or rescan. mRNA decay is determined by Poly(A) shortening and could also depend on codon occupancy. The Poly(A) shortening is the focus of interest here. Poly(A) shortening is subject to the removal of the PABP which is further controlled by the total initiation rate.

As mentioned above, TASEP type mean-field models can be used to facilitate the study on such systems,

which consists of n equations for the translation process coupled with a equation for PAB removal, as follows,

$$\begin{cases} \dfrac{dx_1}{dt} =\alpha_0(1-x_1)-\gamma_1 x_1(1-x_2) \\ \dfrac{dx_2}{dt} =\gamma_1 x_1(1-x_2)-\gamma_2 x_2(1-x_3) \\ \ldots=\ldots \\ \dfrac{dx_s}{dt} =\gamma_{s-1}x_{s-1}(1-x_s)-(1-\delta_{PT})\gamma_s x_s(1-x_{s+1})-\delta_{PT}\gamma_s x_s \\ \dfrac{dx_{s+1}}{dt} =(1-\delta_{PT})\gamma_s x_s(1-x_{s+1})-\gamma_{s+1}x_{s+1}(1-x_{s+2}) \\ \ldots=\ldots \\ \dfrac{dx_n}{dt} =\gamma_{n-1}x_{n-1}(1-x_n)-\beta x_n \\ \dfrac{dN_B}{dt} =-k_B \end{cases} \tag{1}$$

For the translation process which is composed of the first n equations, x_i depicts the coverage probability of codon i, s is the early PTC codon, α_0 is the natural initiation rate, β is the termination rate at the normal stop codon, $\gamma_i, i=1,2\ldots,n-1$ is the elongation rate of codon i, and δ_{PT} is the premature termination rate at codon s. For the last PAB removal equation, N_B is the number of PABs, and k_B is the decay rate for PABs given by

$$k_B=\frac{k_{B0}}{k_I} \tag{2}$$

where k_{B0} is the natural removal rate and k_I is the total initiation rate. This describes in a succinct way the negative correlation between the PABP removal rate and the initiation rate. For the basic model structure shown in Fig. S1, $k_I=\alpha_0$, but in the numerical calculation of general PABP removal model together with the mean-field TASEP model, the total initiation rate k_I is time varying since k_I is affected by other dynamic factors such as the ribosome recycle rate, as seen in the later model which considers ribosome recycle.

The stochastic model involves a standard stochastic description incorporating mRNA translation dynamics, PABP removal (connected to initiation) and the concomitant effect on the mRNA stability all described stochastically. The simulation methods used here are based on [1]. The PABP removal process in stochastic simulations is interpreted as follows [2, 3]: within small time t the probability that the rightmost PABP still remains there is given by $e^{-k_B t}$, or equivalently, the probability of the rightmost PABP being removed during time t is given by $1-e^{-k_B t}$.

Hence, the initiation event has the effect of slowing down the removal process of the PABP. If we suppose Poly(A) is shortened immediately after PAB removal and mRNA decays immediately after all PABP are

removed, the average time for mRNA decay can be written as

$$T = N_B \frac{1}{k_B} = \frac{N_B}{k_{B0}} k_I \quad (3)$$

That is, under the assumptions, k_I can be a reasonable estimate of the average decay time of the mRNA.

Parameter values for these models and others models, are discussed in Section 3.

2.2 Codon-based model with recycle

The next model in the hierarchy is the codon-based model incorporating recycle. The model structure is shown in Fig. S2. As depicted in the figure, ribosome recycle from both the premature and normal stop codons are included. For each of the stop codons, there is a probability (factor δ_s for the premature stop codon and factor δ_n for the regular termination codon) that the terminating ribosome may be recycled. This probability may also depend on the location of the premature termination codon.

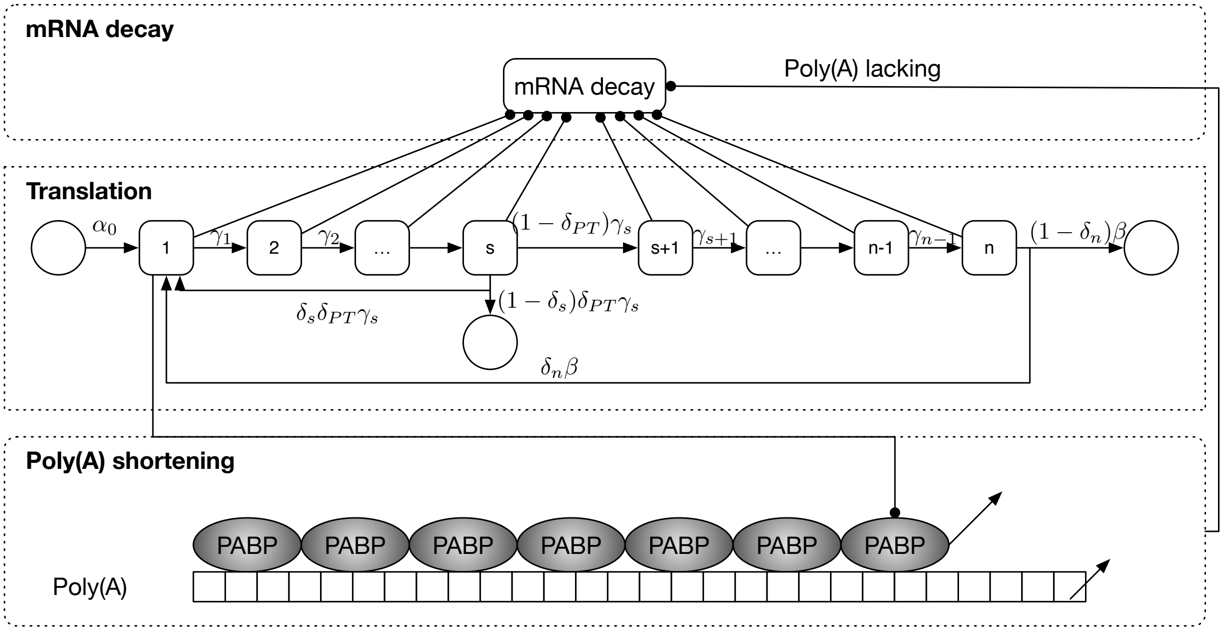

Figure S2: The general codon-based model structure without ribosome rescan. Poly(A) shortening is subject to the removal of the PABP which is further controlled by the total initiation rate. The total initiation rate consists of the de novo rate α_0, and the recycled from the PTC and the NTC.

Again the events in Fig. S2 can be described by a mean-field TASEP like model, which simply augments the corresponding model presented previously, with the recycle events, where the variables are defined in the

basic model.

$$
\begin{cases}
\frac{dx_1}{dt} =k_I(1-x_1)-\gamma_1 x_1(1-x_2)\\
\frac{dx_2}{dt} =\gamma_1 x_1(1-x_2)-\gamma_2 x_2(1-x_3)\\
\quad \ldots =\ldots\\
\frac{dx_s}{dt} =\gamma_{s-1}x_{s-1}(1-x_s)-(1-\delta_{PT})\gamma_s x_s(1-x_{s+1})-\delta_s\delta_{PT}\gamma_s x_s(1-x_1)-(1-\delta_s)\delta_{PT}\gamma_s x_s\\
\frac{dx_{s+1}}{dt} =(1-\delta_{PT})\gamma_s x_s(1-x_{s+1})-\gamma_{s+1}x_{s+1}(1-x_{s+2})\\
\quad \ldots =\ldots\\
\frac{dx_n}{dt} =\gamma_{n-1}x_{n-1}(1-x_n)-(1-\delta_n)\beta x_n-\delta_n\beta x_n(1-x_1)\\
\frac{dN_B}{dt} =-k_B
\end{cases}
\tag{4}
$$

Note here that k_B is still in the form of (2) but with different total initiation rate $k_I = \alpha + \delta_n \beta x_n + \delta_s \delta_{PT} \gamma_s x_s$.

Just as before the entire sequence of events in the model can be described stochastically. What makes the difference from the basic deterministic model in (1) is that the calculation of k_I requires the coverage densities x_s and x_n to be known, but in stochastic simulations each codon has only two states, either covered or not, and this changes stochastically. In order to reflect the correlation between initiation rate and the PABP removal stochastically, we numerically average the effective initiation rate (using the stochastic simulations) over a small time interval i.e., all ribosome coverage instances of the considered codon during the time interval are summed first, and then the average coverage density is obtained by averaging the sum over this interval. In this manner we reflect the correlation between initiation rate and PABP removal so that a (local) average initiation rate (which incorporates some time averaging intrinsic to the biological process) is the input to the PABP removal. This time interval is small relative to the time scale of simulations, but allows for local temporal averaging of the stochastically varying codon coverages and initiation. Our results are insensitive to the particular choice of the time interval, and our stochastic simulation results mirror both the results observed with the deterministic model, while also replicating patterns observed experimentally.

2.3 Codon-based model with ribosome rescanning

The models in the previous section employ a basic description of mRNA translation overlaid by PABP removal, as well as premature termination and recycle. As such the basic model structure is similar to those employed in the literature. Our study of the destabilization of the early premature termination codon led us

to examine the possibility of ribosomal rescanning (which is admissible for this location). To the best of our knowledge, ribosomal rescanning has not been modelled in detail in the literature. Our model involves an augmentation of the earlier models to incorporate this key feature. Here the modelling is purely stochastic.

The modelling of ribosomal rescan involves incorporating the key features of rescan. A terminated ribosome at the premature stop codon can rescan, continue on the mRNA at a different rate (depicted B, in Figure 3), reinitiate downstream in any of the three reading frames, and progresses till the premature stop codon in this frame, where another termination and rescanning event happens, leading to a ribosomal fragment progressing till it reaches another initiation codon, whereupon it is completed, and progresses in the downstream zero frame and is terminated in the regular termination codon.

We have employed two levels of computational models which describe this process (Fig. S3 and Fig. S4) which differ in the level of detail in the stochastic simulation. We include both for the sake of completeness, and note at the outset that essentially similar results were obtained from both models. The less detailed way is described in Fig. S3 and the essential difference is that while it incorporates ribosomal rescanning, it does not incorporate the difference between the zero frame and the +1 frame, or the fact that the ribosomal unit progresses at a different rate.

- **The simple way.** Using this model the +1 frame start and stop codons are treated approximately as the nearby zero frame codons, and the rescanning ribosome moves codon by codon. The model structure is illustrated in Fig. S3.

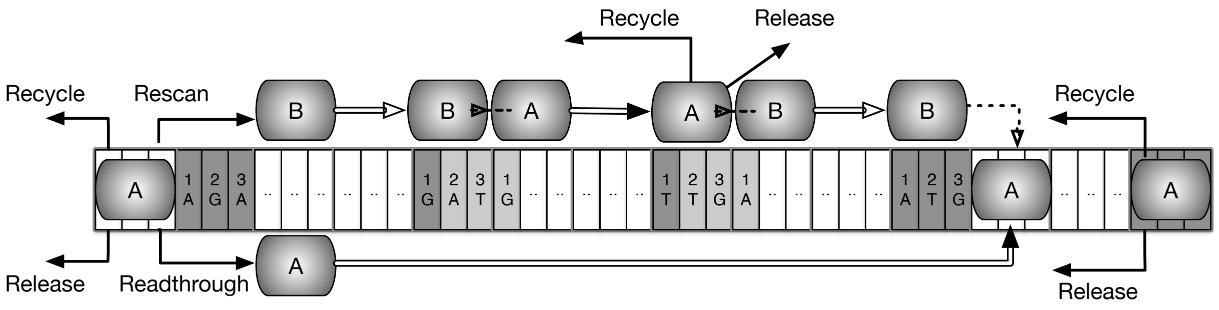

Figure S3: Modelling ribosome rescan: the simple way. At the early PTC (C22), the ribosome can either terminate and recycle, or terminates and releases, or terminates and rescans, or reads through. Between the early PTC and the downstream zero frame PTC, the readthrough ribosome moves normally (labeled as type A) while the rescan ribosome (labeled as type B) may move at different rate. At the downstream +1 frame start codon, the type B ribosome will change to type A and soon change back to type B at the next in-frame stop codon. Finally downstream type B ribosome changes to type A and continues to translate till the end. In this model the +1 frame start and stop codons are treated as the zero frame codons.

- **The detailed way.** The detailed model incorporates additional details not present in the simpler model. Here, we describe the codons between the first PTC and the downstream zero frame PTC in much more detail, in fact transitioning from codon based to nucleotide based descriptions. Type A ribosome will move per codon but type B progresses per nucleotide. Strict exclusion is enforced. From the viewpoint of a computational model, the primary changes are: 1) the description of the concerned codons; 2) the calculation of the possible events and the corresponding event rates, which arise from this detailed description. The logic of the ribosome movement of this detailed description can be seen in Algorithm 1, with the model structure being illustrated in Fig. S4 for details.

Algorithm 1 Modelling ribosome rescan: the detailed way.

---

§1. A ribosome (labelled type A) initiates its translation and moves along the mRNA until it reaches the early PTC codon. The next move can be either of the following four stochastic events:

- Termination and recycle. This increases the initiation rate.
- Rescan. The ribosome is labelled type B.
- Released to the pool.
- Readthrough. This type A ribosome then moves normally.

§2. The newly labelled type B ribosome (if it rescans) moves along the ribosome at a different progression rate, nucleotide by nucleotide.

- It is changed to type A ribosome at the head of the downstream start codon and continues progress, codon by codon;
- At the next in-frame stop codon it either rescans and changes back to type B ribosome, or recycles, or is released (no readthrough).
- The rescanning type B ribosome moves again nucleotide by nucleotide until it reaches the head of the downstream PTC, then it changes back to type A ribosome and moves codon by codon normally.

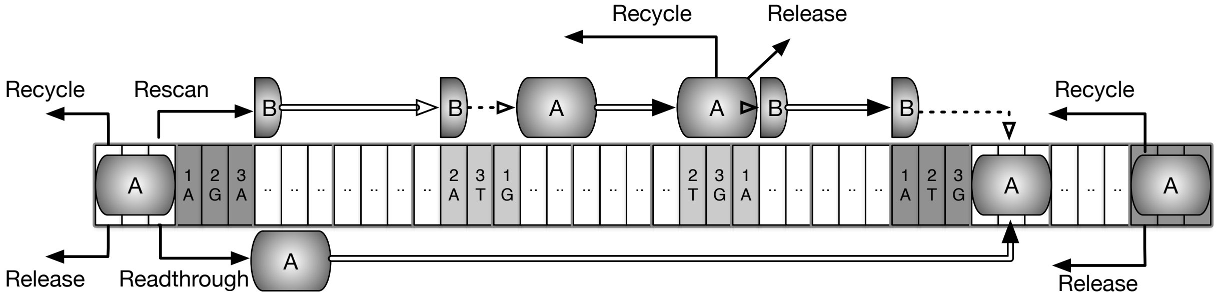

Figure S4: Modelling ribosome rescan: the detailed way. At the early PTC (C22), the ribosome can either terminates and recycles, or terminates and releases, or terminates and rescans, or reads through. Between the early PTC and the downstream zero frame PTC, the readthrough ribosome moves normally (labeled as type A) while the rescan ribosome (labeled as type B) can move per nucleotide and therefore it can reach the next in-frame start codon. Then the type B ribosome will reinitiate and change to type A and soon change back to type B at the downstream stop codon. Finally at the downstream zero frame PTC, the type B ribosome changes to type A and continues to translate till the end.

3 Simulation studies

3.1 Parameters

The core of the model involves the translation process as well as the effect on mRNA stability through poly(A) shortening. For the mRNA translation, we choose parameters so that elongation rate constants are the same , also the same as termination at the regular codon. The initiation rate constant is factor of ten less than the elongation rate constant: this is consistent with many experimental studies that initiation is a rate limiting step in mRNA translation. For the premature termination codon, we have examined two cases: one where the termination rate constant is clearly less than the elongation/ regular termination rate constants (by a factor of ten), and another where the termination rate constant at the premature stop codon is the same as that of the regular termination constants.

Our simulation involves a coarse grained description of the mRNA into 35 segments, with the location of the premature termination codons determined from the experimental context. This coarse grained description is sufficient for our analysis as it reveals the key sources which promote destabilizing of the mRNA: (a) reduced recycle or (b) queueing due to slow termination which reduces new initiation. Both these factors are at play even with a detailed description of the ribosomal progression on the mRNA including additional details such as ribosomal coverage of multiple codons, and a comprehensive codon-based description of the mRNA. Our model is motivated directly by experiments, and is coarse grained to provide basic insights for this context. Furthermore the model makes predictions and conclusions which mirror what are observed experimentally. Consequently it is an adequate representation from the focal point of the study.

Specifically, the parameters used in the simulations are chosen as follows. The mRNA contains 35 segments, single coverage of the ribosome, base initiation rate of 0.1 and elongation and termination rates being 1. We consider 5 different PTC positions, on codons 2, 12, 19, 27, 33, respectively. The reader may notice that the PTC at codon 2 is used to resemble the C22 early PTC in real translation, and other PTCs are presented for comparison purposes. The total rate at PTC $\gamma_s = 1$, the recycle propensity at normal stop codon $\delta_n = 0.9$, the PAB removal rate $k_{B0} = 0.01$, and the number of PABP is 30. The recycle propensity at all premature termination codons is 0.9, except the earliest codon, where it is assigned a value 0.2. We have studied two parameter regimes for the premature termination codon: one where the termination rate constant is the same as that of the regular termination codon (non-queueing regime) and one where it is a factor of ten less that that of the regular termination (and elongation) rate constants (queueing regime). The latter is shown in the main text, while both sets are shown together and discussed briefly below. In the case of the rescan simulations, the termination rate constants at additional termination codons, unless

otherwise specified, are the same as those of the regular termination codons, as is the recycle propensity. The rescan propensity is assumed to be 0.3.

Other simulation-specific parameters are listed in the captions of the figures to follow.

3.2 Simulations results

This section presents the simulation results using codon-based models. In Figs. S5 and S6, we perform numerical calculations using TASEP like models, and Fig. S7 shows stochastic simulations, all without ribosome rescan. These simulations study two parameter regimes, the queueing and the non-queueing regimes: the former is shown in the main text, and in the Supplementary Material, we show both these together for the sake of comparison. We see that under our model assumptions, all cases can reproduce the phenomenon as seen in the experiments.

Furthermore in Fig. S8 we show the stochastic simulations with ribosome rescan. We see that in the non-queueing regime, rescan results in a modest increase in stability while in the queueing regime, it introduces a small decrease in stability at the premature stop codon. Noting the trends associated with different stop codons seen in previous plots, we can conclude that the same trends are maintained, even with the possibility of rescan (at the earliest PTC). Note that in the queueing regime, having rescan introduces addition termination codons for the rescanned ribosomes, which are assumed to have the same rate constant as the PTC, introducing additional queueing effects. For the sake of completeness in Fig. S8(e) and Fig. S8(f), we show rescan borrowing from recycle. Thus we compare cases with non-trivial amounts of recycle (with no rescan) with analogous cases, where rescan borrows from recycle. Rescan has a destabilizing effect.

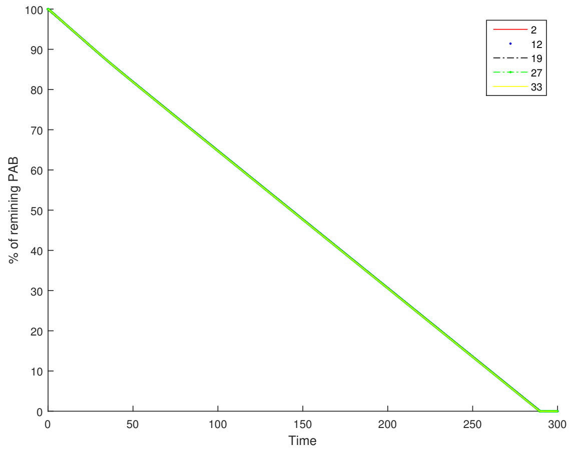

(a) No recycle at premature stop codons. Position has very little effect on stability.

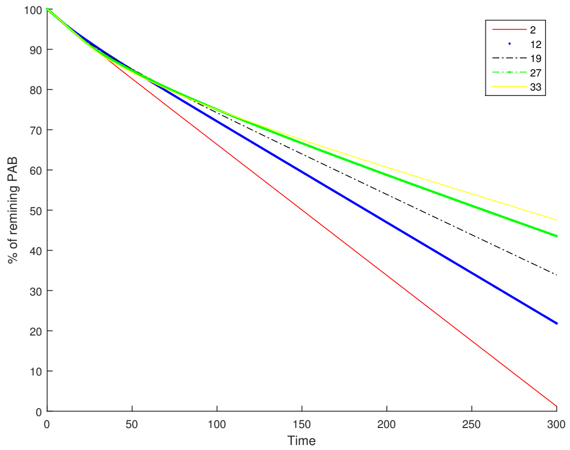

(b) Linearly increasing recycle propensity results in a gradation of stability: earliest stop codon is least stable. Recycle probability is linearly increasing with PTC position.

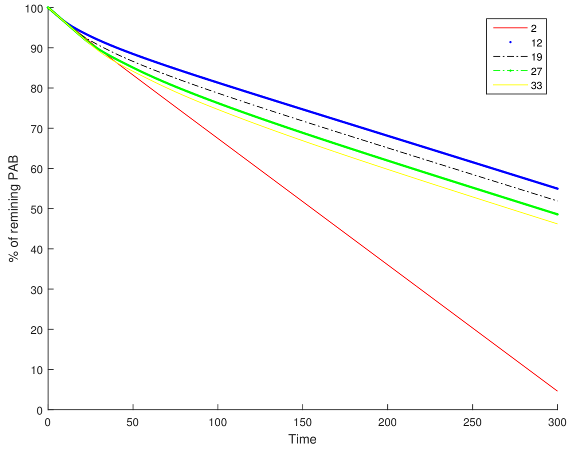

(c) Recycle with a sharp switch results in a sharp gradation in stability (similar to data). Recycle propensity is 0.1 for codon 2 and 0.9 for all others.

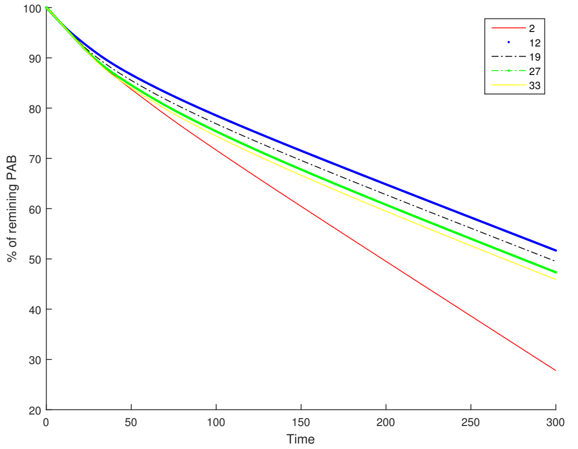

(d) Moderate readthrough (50%) does not change the essential pattern of the previous case.

Figure S5: This figure deals with the non-queueing regime. There is recycle at the regular stop codon. The elongation and termination rate constants are the same, as is the overall rate constant at the premature termination codon. The de-novo initiation rate constant is one-tenth the other rate constants. The readthrough propensity is 0.1 for (a)(b)(c) and 0.5 for (d). In the above, recycle contributes significantly to the overall initiation. Consequently, a gradation in recycle can directly explain the trends observed. An increase in readthrough has a moderate effect of increasing the stability of the earliest stop codon, while the overall trends are maintained.

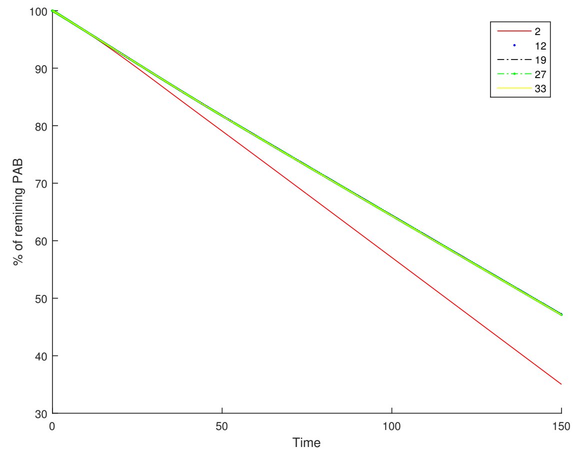

(a) The position of the premature stop codon can play an important role: the earliest stop codon has a reduced stability compared to the other 4 (whose curves are overlaid upon one another). No recycle in all the premature stop codons.

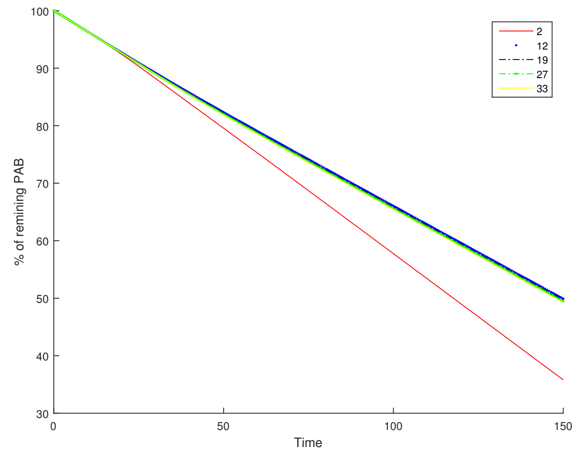

(b) Adding uniform recycle at premature stop codons maintains the essential pattern seen previously. Recycle propensity is 0.1 for all.

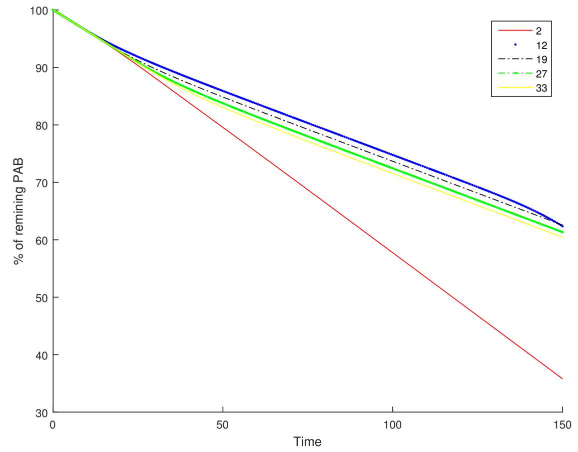

(c) A sharply increasing recycle propensity (analogous to the case considered previously) can still result in similar trends. Recycle propensity is 0.1 for codon 2 and 0.9 for all others.

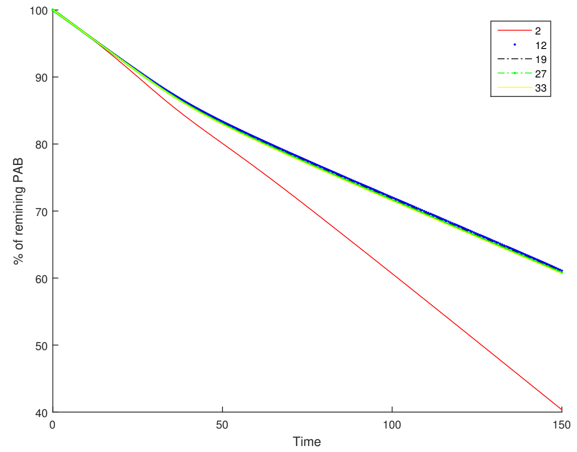

(d) Theoverall pattern is insensitive to readthough (90%).

Figure S6: This figure deals with the queueing regime, simulated exactly as in Fig. S5. Here the elongation and regular termination rate constants are the same which is 10 times as high as the de-novo initiation rate constant and the total rate constant at the premature termination codon. Note that decreasing the total rate constant at the premature stop codon can accentuate the queueing effect. Readthrough propensity is 0.1 for (a)(b)(c) and 0.9 for (d). Note that in (d) we have increased the read through propensity keeping the total rate constant at the premature termination codon fixed.

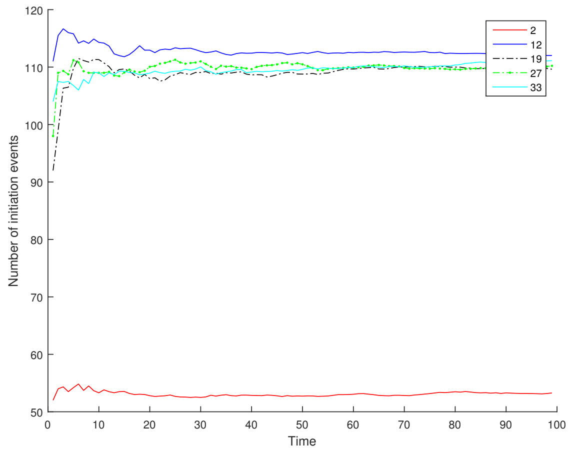

(a) Non-queueing regime (see Fig. S5). Recycle propensity is 0.1 for codon 2 and 0.9 for all others.

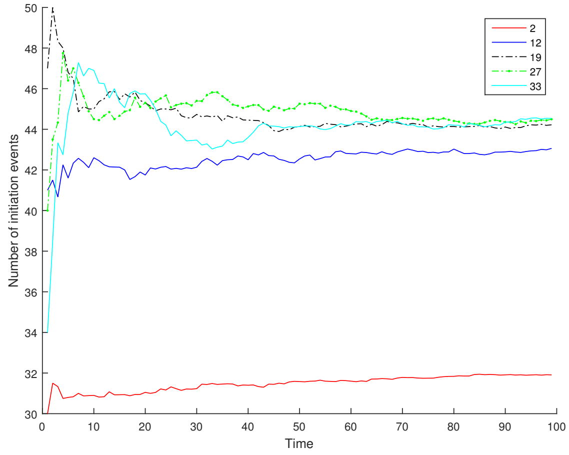

(b) Queueing regime, no recycle (analogous to Fig. S6(a)).

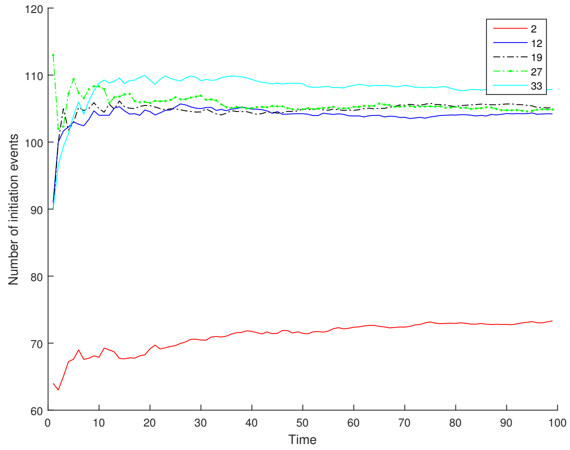

(c) Non-queing regime above in (a) with high readthrough (50%)

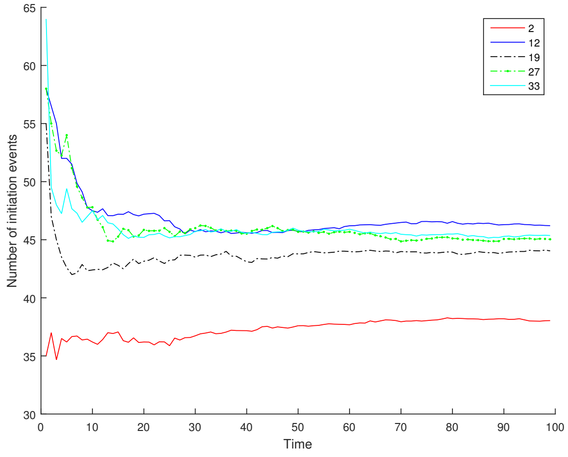

(d) Queueing regime above in (b) with high readthrough (90%)

Figure S7: Stochastic simulations show similar patterns of stability as those seen in Figs. S5 and S6.

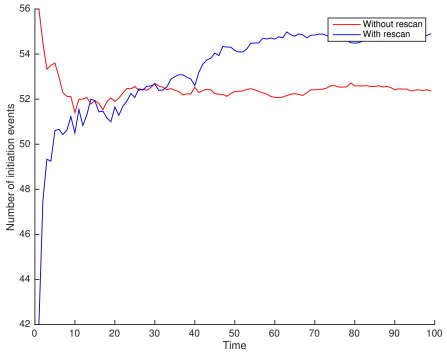

(a) Non- queuing regime, with sharply increasing recycle as in Fig. S5(c)

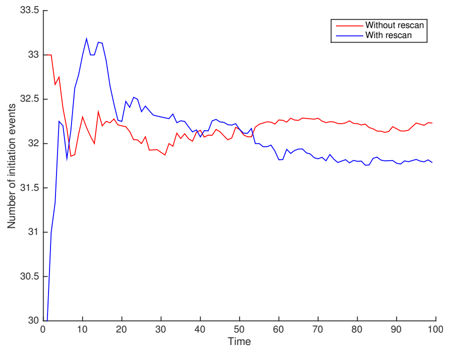

(b) Queuing regime with no recycle as in Fig. S6(a)

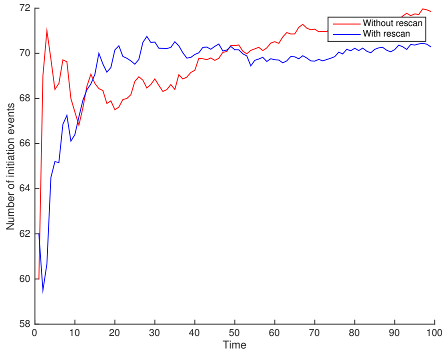

(c) Non-queing regime with high readthrough, as in Fig. S5(d)

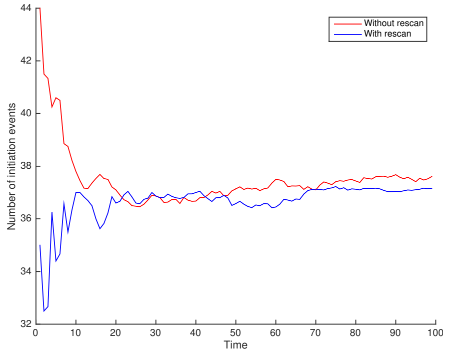

(d) Queueing regime with high readthrough as in Fig. S6(d)

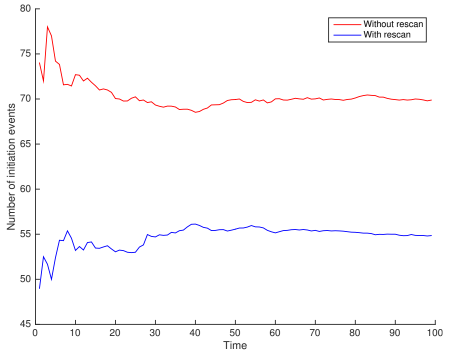

(e) Non-queueing regime. Rescan directly borrows from recycle.

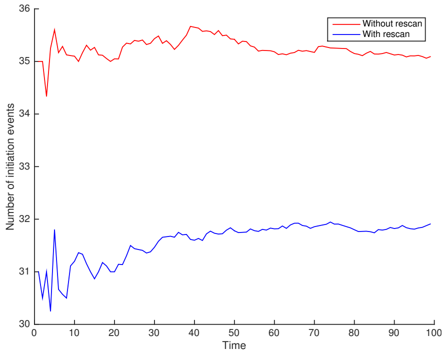

(f) Queueing regime: Rescan directly borrows from recycle.

Figure S8: This figure explores the effect of rescan at the earliest stop codon, contrasted with the case of no rescan, using stochastic simulations. The left column and the right column are for the non-queuing and queuing regimes, respectively. Rescan propensity is 0.3 in all cases. In (a), (b), (c),(d) rescan is introduced so as to take away from release into the pool.

References

1. Zhao Y.-B, Krishnan J: **mRNA translation and protein synthesis: an analysis of different modelling methodologies and a new PBN based approach**. *BMC Syst. Biol.* 2014, **8**(1):25.
2. Muhlrad D, Decker CJ, Parker R: **Turnover mechanisms of the stable yeast PGK1 mRNA**. *Mol. Cell. Biol.* 1995, **15**(4):2145–2156.
3. Schwartz DC, Parker R: **Mutations in Translation Initiation Factors Lead to Increased Rates of Deadenylation and Decapping of mRNAs in Saccharomyces cerevisiae**. *Mol. Cell. Biol.* 1999, **19**(8):5247–5256.
4. Rajkowitsch L, Vilela C, Berthelot K, Ramirez CV, McCarthy JE: **Reinitiation and recycling are distinct processes occurring downstream of translation termination in yeast**. *J. Math. Biol.* 2004, **335**:71–85.

Supplementary information
Table S1

Primer designation	Sequence (5’-3’)
1099	CAAAAGGTCAAGGCTTCCTAAGAAGATGTTCAAAAGTTCAG
1100	CTGAACTTTTGAACATCTTCTTAGGAAGCCTTGACCTTTTG
1101	GCTGACAAGATTCAATTGATTGACAACTAAGTGGACAAGGTCGACTCT
1102	AGAGTCGACCTTGTCCACTTAGTTGTCAATCAATTGAATCTTGTCAGC
1103	GTTGGACAATGGTCCAGAATCTTAAGAGTTGTTTGCTGCTACTGTT
1104	AACAGTAGCAGCAAACAACTCTTAAGATTCTGGACCATTGTCCAAC
1168	GGGACGTTGAAGTCAACCTAGATGAAGACACGCTT
1169	AAGCGTGTCTTCATCTAGGTTGACTTCAACGTCCC
1202	TGGGACGTTGAAGTCAACCTATTGGAAGACACGCTTGTCCTTCAA
1203	TTGAAGGACAAGCGTGTCTTCCAATAGGTTGACTTCAACGTCCCA
1204	GTCCAATGGGACGTTGAAGTCTTGCTATTGGAAGACACGCTTGTC
1205	GACAAGCGTGTCTTCCAATAGCAAGACTTCAACGTCCCATTGGAC
1076	GGAATTCCCCCCCCCCCCCCCCCCAAATTCC
1096	GGTGGTGGTGACACTGCC
1113	GTGGGATGGGATACGTTGAG
1114	GTGTCTAGCCGCGAGGAA

Table S1: Primers used in this study
